# Barriers and facilitators to implementation of evidence-based task-sharing mental health interventions in low- and middle-income countries: a systematic review using implementation science frameworks

**DOI:** 10.1186/s13012-021-01179-z

**Published:** 2022-01-12

**Authors:** PhuongThao D. Le, Evan L. Eschliman, Margaux M. Grivel, Jeffrey Tang, Young G. Cho, Xinyu Yang, Charisse Tay, Tingyu Li, Judith Bass, Lawrence H. Yang

**Affiliations:** 1 Department of Social and Behavioral Sciences, New York University School of Global Public Health, 708 Broadway, NY 10012 New York, USA; 2 Department of Health, Behavior and Society, Johns Hopkins University Bloomberg School of Public Health, 615 North Wolfe St., Baltimore, MD 21205 USA; 3 Department of Psychology, New York University Graduate School of Arts and Science, One-Half Fifth Avenue, New York, NY 10003 USA; 4 New York State Psychiatric Institute, Columbia University, 1051 Riverside Dr., New York, NY 10032 USA; 5 Department of Epidemiology, Columbia University Mailman School of Public Health, 722 West 168th St., New York, NY 10032 USA; 6 Columbia University Teachers College, 525 West 120th Street, New York, NY 10027 USA; 7 Department of Mental Health, Johns Hopkins University Bloomberg School of Public Health, Hampton House, 8th Floor, 624 N. Broadway, Baltimore, MD 21205 USA

**Keywords:** Task-sharing, Mental Health, Evidence-based practices, Barriers and facilitators, Low- and middle-income countries, Implementation strategies

## Abstract

**Background:**

Task-sharing is a promising strategy to expand mental healthcare in low-resource settings, especially in low- and middle-income countries (LMICs). Research on how to best implement task-sharing mental health interventions, however, is hampered by an incomplete understanding of the barriers and facilitators to their implementation. This review aims to systematically identify implementation barriers and facilitators in evidence-based task-sharing mental health interventions using an implementation science lens, organizing factors across a novel, integrated implementation science framework.

**Methods:**

PubMed, PsychINFO, CINAHL, and Embase were used to identify English-language, peer-reviewed studies using search terms for three categories: “mental health,” “task-sharing,” and “LMIC.” Articles were included if they: focused on mental disorders as the main outcome(s); included a task-sharing intervention using or based on an evidence-based practice; were implemented in an LMIC setting; and included assessment or data-supported analysis of barriers and facilitators. An initial conceptual model and coding framework derived from the Consolidated Framework for Implementation Research and the Theoretical Domains Framework was developed and iteratively refined to create an integrated conceptual framework, the Barriers and Facilitators in Implementation of Task-Sharing Mental Health Interventions (BeFITS-MH), which specifies 37 constructs across eight domains: (I) client characteristics, (II) provider characteristics, (III) family and community factors, (IV) organizational characteristics, (V) societal factors, (VI) mental health system factors, (VII) intervention characteristics, and (VIII) stigma.

**Results:**

Of the 26,935 articles screened (title and abstract), 192 articles underwent full-text review, yielding 37 articles representing 28 unique intervention studies that met the inclusion criteria. The most prevalent facilitators occur in domains that are more amenable to adaptation (i.e., the intervention and provider characteristics domains), while salient barriers occur in domains that are more challenging to modulate or intervene on—these include constructs in the client characteristics as well as the broader societal and structural levels of influence (i.e., the organizational, mental health system domains). Other notable trends include constructs in the family and community domains occurring as barriers and as facilitators roughly equally, and stigma constructs acting exclusively as barriers.

**Conclusions:**

Using the BeFITS-MH model we developed based on implementation science frameworks, this systematic review provides a comprehensive identification and organization of barriers and facilitators to evidence-based task-sharing mental health interventions in LMICs. These findings have important implications for ongoing and future implementation of this critically needed intervention strategy, including the promise of leveraging task-sharing intervention characteristics as sites of continued innovation, the importance of but relative lack of engagement with constructs in macro-level domains (e.g., organizational characteristics, stigma), and the need for more delineation of strategies for task-sharing mental health interventions that researchers and implementers can employ to enhance implementation in and across levels.

**Trial registration:**

PROSPERO CRD42020161357

**Supplementary Information:**

The online version contains supplementary material available at 10.1186/s13012-021-01179-z.

Contributions to the literature

- Task-sharing based on evidence-based practices is a promising strategy to increase the global availability of mental health care, and no literature has systematically compiled barriers and facilitators to implementation of these interventions in low- and middle-income countries.
- Barriers and facilitators to evidence-based task-sharing mental health interventions are present at multiple levels, and we provide a conceptual framework to organize them into eight domains.
- Our enumeration and organization of the implementation barriers and facilitators is an important step in helping practitioners and researchers improve the implementation of these interventions, including the development of tools to measure these implementation determinants and the need to select implementation strategies that can specifically leverage the facilitators and target the barriers across multiple levels.

## Introduction

As a key part of global mental health efforts to address the mental health treatment gap, increasingly widespread adoption of task-sharing strategies has led to significant expansions of the mental healthcare workforce and improvements in population mental health and well-being globally [1–3]. Task-sharing involves the formalized redistribution of care typically provided by those with more specialized training (e.g., psychiatrists, psychologists) to individuals, often in the community, with little or no formal training (e.g., community/lay health workers, peer support workers). Task-sharing mental health interventions have been primarily implemented and evaluated in low- and middle-income countries (LMICs) [4], where the mental health treatment gap is most severe. In LMICs, estimates suggest that 75% of individuals who require mental health treatment are not receiving appropriate services due to a variety of barriers—including the scarcity of mental health specialists [5]. Models of task-sharing mental health interventions are varied, such as utilizing primary care providers to detect or provide care for mental health concerns within a broader healthcare system [6–8]; training and supervising community/lay health workers to administer psychotherapy for common mental disorders [6, 9]; and employing service users themselves (e.g., as peer support workers) to augment mental health interventions [10–13].

There is growing interest in further understanding the development, implementation, and outcomes of the task-sharing approach. One element of this growing field of research is the examination of implementation determinants, commonly referred to as barriers and facilitators*,* defined by Lewis et al. (2018, p. 2) as “factor[s] that enable or hinder the implementation strategy from eliciting the desired effect” [14]. For example, researchers are examining whether implementation determinants can help explain why the peer-delivered Thinking Healthy Programme demonstrated modest benefits on perinatal depression in India, but no significant impacts in Pakistan [15, 16]. Similarly, a research team in Colombia is exploring what type of implementation factors can explain some of the variance in effect sizes across project sites in a trial of a transdiagnostic cognitive behavioral intervention provided by lay health providers [17]. An important part of these efforts to understand implementation determinants of task-sharing mental health intervention strategies is the ability to systematically categorize the identified factors to then inform targeted adjustments to implementation strategies that can optimize the intervention effects [18, 19].

While there have been studies and reviews that have discussed barriers and facilitators to task-sharing mental health interventions, they have limited their search and analyses to specific settings, (e.g., rural areas in high-income countries, [20]), specific mental health conditions (e.g., perinatal depression, [12]), populations (e.g., youth mental health, [21]), or task-sharing models (e.g., integration into primary care, [22]). Additionally, these and other reviews (for example, see [23]) have not been explicitly situated in an implementation science framework, limiting the applicability of the results to other task-sharing interventions, models, or settings.

The purpose of this review is to use an implementation science perspective to identify barriers and facilitators to the implementation of evidence-based task-sharing mental health interventions focusing on LMICs, as task-sharing has been most widely used in these low-resource settings.

The specific research questions and objectives for the review were:

1. What are the reported barriers and facilitators in the implementation of evidence-based task-sharing mental health interventions in LMICs?
2. How can implementation science frameworks and constructs be used to guide the understanding of the implementation determinants of task-sharing mental health interventions?
3. How do these barriers and facilitators inform the selection and further study of implementation strategies for task-sharing mental health interventions?

## Methods

### Protocol, registration, reporting guidelines

The protocol was registered with The National Institute of Health Research’s international prospective register of systematic reviews (PROSPERO CRD42020161357). This systematic review was conducted using the Preferred Reporting Items for Systematic Review (PRISMA) guidelines [24], and the PRISMA checklist is provided in Additional file 1A: PRISMA Checklist.

### Search strategy

We conducted searches using PubMed, PsychINFO, CINAHL, and Embase to identify peer-reviewed studies published before July 1st, 2019. Studies from all years up until July 1st, 2019 were included to be as comprehensive as possible. Terms were compiled for three broad categories: “mental health,” “task-sharing,” and “LMIC.” “Mental health” and “task-sharing” used search terms from previously published systematic reviews of task-sharing mental health interventions [23, 25, 26] and were edited based on our manual search of key articles in the field. Search terms for LMIC were provided by a public health informationist at the Johns Hopkins Welch Medical Library and supplemented with the list of LMICs from the World Bank [27]. An initial comprehensive search phrase was developed for PubMed (See Additional file 1B: Search Syntax) and the search syntax was adapted for the remaining databases. Searches were restricted to English-language and articles with human subjects. We identified additional studies by reviewing the reference list of recent systematic reviews and meta-analyses.

### Study selection

Title and abstract screening was completed in two phases. In the first phase, the lead and senior authors (PTL and LHY), along with a team of masters-level research assistants, collaboratively screened 200 titles and abstracts to finalize screening protocols and ensure consistency in the screening process. In the second phase, the remaining titles and abstracts were each independently screened by two research assistants, with disagreements resolved by the lead authors and reviewed by the team. A similar process was used for the full-text article review: the lead authors and research assistants collaboratively reviewed 100 full-text articles, then full-text screening was conducted, with each article reviewed by two independent research assistants and discrepancies resolved by the lead authors. We used the software Covidence [28, 29] to manage the screening and review processes.

We then used a two-stage search process to reach our finalized set of articles. Studies were first evaluated against the following inclusion criteria: (1) the major focus of the intervention/study was a mental health condition, defined as common and serious mental health conditions excluding neurological and substance use disorders (2) the intervention/study was implemented in a LMIC; (3) the intervention involved task-sharing/task-shifting; and (4) the research article included direct assessments of barriers and facilitators or analysis and insights drawn from empirical data. Our focus included serious mental health conditions as well as common mental health conditions due to the noted need to expand the evidence base for task-sharing for serious mental health conditions [30]. We did not include neurological disorders and substance use disorders because the task-sharing interventions targeting these conditions are likely to have distinct sets of barriers and facilitators. Task-sharing interventions for neurological disorders such as epilepsy are predominantly focused on pharmacologic aspects (e.g., medication adherence), while interventions for substance use are often integrated into service delivery models for other health conditions such as HIV/AIDS. Additional exclusion criteria were: (1) it was not a research article (i.e., was a dissertation, comment, or review); (2) the peer-reviewed publication was not in English; (3) the intervention did not have a major focus on common and/or serious mental health conditions; (4) the study did not employ a task-sharing strategy; and (5) the study was not conducted in an LMIC. In an additional step, we finalized the set of included studies to include only studies on interventions that used or were based on evidence-based practices (EBPs). No comprehensive, authoritative list of EBPs exists, so treatments were considered EBPs if they had “strong research support” as designated by the American Psychological Association (APA) or were “strongly recommended” by APA or the World Health Organization (WHO). In this additional phase of screening, studies were included if (i) the treatment used in the intervention was an EBP (e.g. interpersonal therapy [IPT], pharmacological treatment), or (ii) the content of the treatment used in the intervention was based on an EBP (e.g., using Cognitive Behavioral Therapy (CBT) principles, using the World Health Organization (WHO) Mental Health Gap Action Program (mhGAP)). Studies excluded at this stage used strategies that have not yet met these designations (e.g., strategies with not fully developed evidence bases), used strategies that were developed specifically for the local context without a foundation in EBPs or did not specify their strategy’s rationales or foundations.

### Development of conceptual model and codebook

Although many conceptual models and meta-frameworks exist in implementation science (for a searchable online repository, see [31]) none has centered on the unique dynamics associated with the task-sharing approach, such as the interactions between clients and the task-sharing providers, and how certain intervention characteristics (e.g., training and supervision) influence these interactions. Thus, we sought to develop a novel, conceptual framework to both guide and be refined by our systematic review.

Our preliminary conceptual model integrated the domains and constructs specified in the Consolidated Framework for Implementation Research (CFIR; [32]) and the Theoretical Domains Framework (TDF; [33]). Both the CFIR and the TDF are meta-frameworks that focus on identifying and unifying implementation determinants from other implementation science models into organized domains. The CFIR consolidates constructs across five domains: (1) characteristics of the individual, (2) intervention characteristics, (3) inner setting, (4) outer setting, and (5) process. The TDF organizes constructs across 14 domains, ranging from individual knowledge and skills to environmental context and social influences.^1^ While the CFIR includes many intervention, organizational, and contextual factors, the TDF further specifies individual-level characteristics that are especially applicable for the task-sharing format due to its focus on provider-led behavioral interventions. Thus, we combined constructs from both frameworks to capture the breadth of implementation determinants that span the individual, intervention, organizational, and contextual levels [34]. In addition, we drew on the multi-level framework from Chaudoir et al. (2013), which posits that implementation outcomes are predicted by five factor levels: (1) client characteristics; (2) provider characteristics; (3) the innovation (i.e., the intervention or evidence-based practice); (4) organization attributes; and (5) structural-level factors [35]. The Chaudoir et al. [35] framework is particularly helpful for task-sharing because it explicitly includes the characteristics of the providers, which have been found to influence implementation and intervention (i.e., client and service) outcomes [10, 36–38].

Using an initial conceptual framework, we developed a preliminary codebook composed of constructs from the CFIR and the TDF. The lead authors trained research assistants to identify and code texts of each included article as belonging to a specific construct and construct grouping (i.e., domain), and as a barrier, a facilitator, or both a barrier and facilitator. Each included article was coded by two research assistants. During the first round of analysis, pairs of research assistants independently coded articles by highlighting and commenting on the text in each article that pertained to a particular construct. The domain, construct, and whether the construct was present as a barrier, a facilitator, or both, was included in each comment. After coding, the pairs reviewed these codes for discrepancies. Group discussions were then held with the lead authors and research assistants to discuss how constructs were or were not manifested in the articles, to resolve discrepancies, and to reach a consensus on definitions for each construct. This process of independent coding, pair review, and group discussion was then repeated until the codebook and its definitions were finalized. All included articles were then recoded by the same pairs with the finalized codebook, and coded text from the articles was extracted into a shared document and sorted into the corresponding domain and construct. Any remaining disagreements were discussed by the team and resolved by the lead authors.

In the second round of analysis, codes that were conceptually related were combined to form new overarching constructs. We also allowed for open coding and created new codes for constructs that were not captured in the existing codebook (e.g., stigma-related constructs). The process of refining and applying the analytical framework was repeated until the research team determined that we had reached the most parsimonious codebook, which formed the final BeFITS-MH framework.

**Fig. 1:**
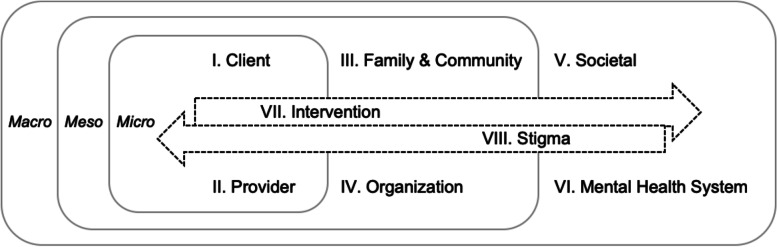
The Barriers and Facilitators in Implementation of Task-Sharing Mental Health (BeFITS-MH) conceptual framework

The resulting conceptual model, the **B**arri**e**rs and **F**acilitators in **I**mplementation of **T**ask-**S**haring **M**ental **H**ealth interventions (BeFITS-MH) framework (Fig. 1), specifies eight nested and intersectional domains across micro-, meso-, and macro-levels. The micro-level domains (Domains I & II) are nested within encompassing meso- and macro-level domains (Domains III & IV and V & VI, respectively) so that individual-level factors can be understood within their immediate (micro-level) and larger contexts (meso- and macro-levels). Specifically, micro-level Client characteristics (Domain I) is nested in the meso-level Family and Community domain (Domain III), which is nested in the macro-level Societal factors (Domain V). Similarly, Provider characteristics (Domain II) is nested in Organizational factors (Domain IV) and Mental Health System factors (Domain VI). The last two domains, Intervention characteristics (Domain VII) and Stigma (Domain VIII), span across the micro-, meso-, and macro-levels and are situated as interactional agents between each other as well as the aforementioned six domains.

Notably, although Chaudoir et al. (2013) situate their “innovation” factor level as interacting primarily with client and provider characteristics, we situate our intervention domain at the nexus of all the domains because intervention characteristics can both influence and be influenced by the other factors—a position also supported by the CFIR [32, 35]. For example, an intervention characteristic such as the role of the task-sharing provider can be affected not only by the individual preferences of the clients and the providers but also by the organizational characteristics (e.g., number of mental health professionals in the clinic) and the societal perceptions about the social status of those employed as task-sharing providers [25]. Furthermore, even though we originally included stigma as a construct of the societal domain, it became apparent in our framework refinement process that, particularly in mental health, stigma (e.g. self-stigma, family/community stigma, provider stigma, organizational or institutional-level stigma) is a force that can exert powerful influences on perceptions of mental health issues and treatments across all social actors and processes—including those in the intervention domain [39–42].

All articles were then re-coded with the final codebook. We then entered the results in a matrix grid presenting domains and constructs in columns, and articles in rows. Of note, although the factors in the mental health system level were conceptualized as distinct from those in the organizational factors domain in that they describe macro-level characteristics that extend beyond a single implementing organization, sometimes factors were coded both in this domain and in the organizational domain if the macro-level issues also exerted influence at the organizational level (e.g., human resources as a barrier at the mental health-system level could also manifest as clinical resources as a barrier at the organizational level). At this stage, we grouped articles derived from the same parent study together before conducting data extraction.

### Data extraction

The following data were extracted into a spreadsheet and cross-checked by two research team members for accuracy: (1) author and publication year; (2) title; (3) country (or countries) where the study occurred; (4) mental health condition(s) addressed; (5) task-sharing model of evidence-based practice; (6) implementation stage(s) about which the barriers and facilitators were referring within the study (i.e., exploration, preparation, implementation, and sustainment, [43]); (7) how the barriers and facilitators were assessed or derived―i.e., directly evaluated by the study or discussed based on intervention data; through qualitative, mixed, or quantitative methods; and with which stakeholder groups; and (8a & 8b) key barriers and facilitators reported, as determined by the analysis process above.

### Quality assessment

All included studies were critically appraised for quality and rigor using the Joanna Briggs Institute’s (JBI’s) critical appraisal tool corresponding to each article’s study type or research design [44, 45]. Of the 37 included articles, approximately half (*n* = 19;) were qualitative studies, while the remaining study designs included RCT (*n* = 8); quasi-experimental (pre-post; *n* = 6); cohort (*n* = 2); and analytical cross-sectional studies (*n* = 2). Some criteria were determined to be unclear for some studies. “NA” was used when the study characteristics rendered certain criteria for the general category of the study design as not applicable, such as the requirement of blinding for a pragmatic trial (for RCTs), or including a reflexivity statement for a process evaluation (for qualitative studies). Among the qualitative studies, all but one met at least 8 of the 10 appraisal criteria. The criteria most frequently unmet included locating the researcher culturally or theoretically (*n* = 9) and addressing the influence of the researcher on the research (*n* = 9). Among the RCTs, none of the included studies had unmet criteria; a few (*n* = 3) had 2–3 unclear criteria. Among the quasi-experimental studies, all met at least 7 of the 9 appraisal criteria. Among the cohort and cross-sectional studies, none had unmet criteria. Overall, over 80% of the included articles had one or fewer unclear criteria, and 95% of the included articles had one or fewer unmet criteria.

As an additional step, the analysis and insights regarding barriers and facilitators in the discussion section of each article was also critically appraised using the three most relevant questions from the JBI’s “Text and Opinion” critical appraisal tool (i.e., “Is the source of the opinion clearly identified?”; “Is the stated position the result of an analytical process, and is there logic in the opinion expressed?”; and “Is any incongruence with the literature/sources logically defended?”) [46]. All articles were met all three of these criteria. Thus, all articles were deemed of sufficiently high quality and were retained (see Additional file 1C).

## Results

### Included studies

**Fig. 2:**
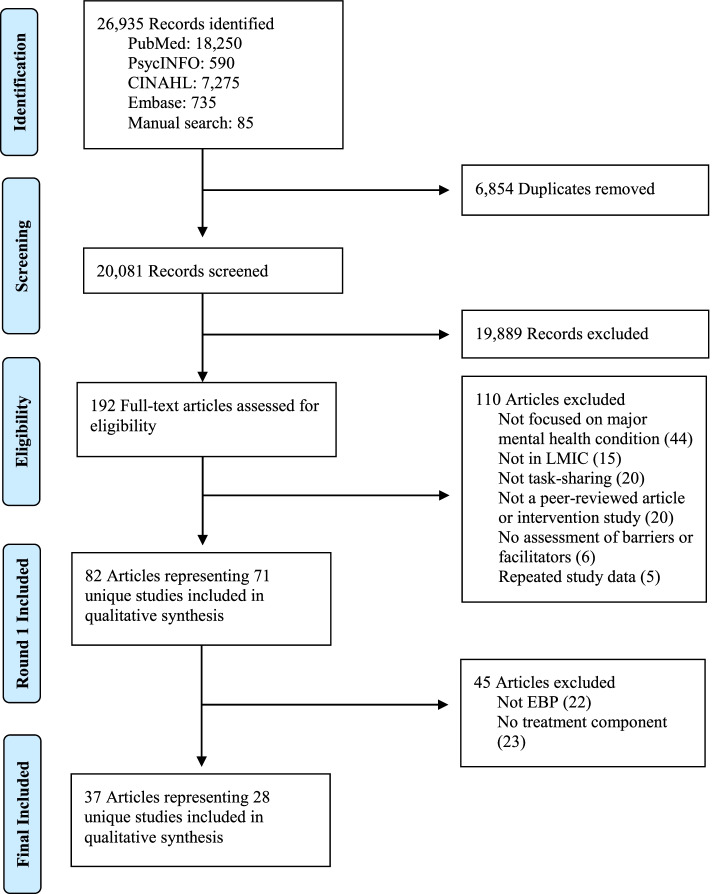
PRISMA flow diagram

A total of 20,081 unique abstracts were identified through database and manual reference searches and underwent title and abstract screening. From this first screening stage, 192 articles received full-text review, with 82 articles (71 unique studies) meeting the initial inclusion criteria. Of the 71 studies initially selected, 28 studies (comprising 37 articles) were determined to use or be based on EBPs using the criteria described above and were selected to comprise the set of studies to be reviewed. See Fig. 2 for the PRISMA flow diagram. The list of 45 studies determined to be not based on EBPs is available from the authors on request.

### Study characteristics

The 28 included intervention studies (from 37 articles) [6, 11, 47–81] were published between 2008 and 2019. Key characteristics of the studies are presented in Table 1. Studies were conducted in 18 LMICs, with the most representation in Africa and South Asia.

**Table 1:** Study and implementation characteristics of *N* = 28 evidence-based task-sharing mental health studies

	***N***	%
*Study characteristics*		
**Region of study**		
Africa	14	50.0%
South Asia	9	32.1%
Latin America and the Caribbean	3	10.7%
Multi-region	2	7.1%
**Mental health condition**		
Common mental disorders (CMDs) only	23	82.1%
*CMDs*	*6*	*21.4%*
*Depression only (non-perinatal)*	*9*	*32.1%*
*Perinatal depression only*	*2*	*7.1%*
*CMDs and comorbid conditions*	*6*	*21.4%*
Serious mental illnesses (SMIs) only	1	3.6%
CMDs and SMIs	4	14.3%
*Implementation characteristics*		
**Type of task-sharing provider (TSP)**		
Community health workers (CHWs)^a^	18	64.3%
Primary health care providers (PHCPs)^b^	4	14.3%
Complementary and alternative providers (CAPs)	1	3.6%
Multiple provider types	5	17.9%
**Evidence-based practice (EBP) used**		
Mental Health Gap Action Programme (mhGAP)	8	28.5%
Cognitive Behavioral Therapy (CBT)	6	21.4%
Interpersonal Therapy (IPT)	3	10.7%
Common Elements Treatment Approach (CETA)	2	7.1%
Pharmacotherapy alone	2	7.1%
Problem-Solving Therapy (PST)	2	7.1%
Problem Management Plus (PM+)	1	3.6%
Multiple EBPs	4	14.3%
**Implementation stage**		
Exploration	1	3.6%
Preparation	4	14.3%
Implementation	17	60.7%
Sustainment	1	3.6%
Multiple Stages	5	17.9%

^a^CHWs include lay health workers, village health workers, lay psychosocial community workers, community health extension workers, community health officers, and community health aids

^b^PHCPs include doctors, nurses, or other healthcare workers in primary care settings

The large majority of studies focused on common mental disorders (CMDs; including depression, perinatal depression, and comorbid conditions), with very few studies reporting on serious mental illnesses (SMIs).

In the majority of studies, the task-sharing providers were community health workers (CHWs; i.e., lay health workers, village health workers, lay psychosocial community workers, community health extension workers, community health officers, and community health aids), followed by primary healthcare providers (PHCPs; i.e., doctors, nurses, or other healthcare workers in primary care settings). Complementary and alternative providers (CAPS; i.e., traditional healers, traditional health practitioners, and faith-based healers) were less represented. Notably, numerous studies utilized multiple task-sharing provider types.

The EBP most commonly used by these studies was mhGAP [82], followed by CBT. Other EBPs used were IPT, the Common Elements Treatment Approach (CETA), pharmacotherapy alone (i.e., studies that did not state that they also used mhGAP or other EBPs to facilitate or promote taking medication), Problem-Solving Therapy (PST), and Problem Management Plus (PM+). Some studies reported the use of multiple EBPs, which usually took the form of a combination of mhGAP guidelines and one of the non-pharmacotherapy treatments listed above or a combination of non-pharmacotherapy treatments. Studies that used mhGAP or non-pharmacotherapy EBPs alongside pharmacotherapy were categorized under their non-pharmacotherapy intervention element.

The large majority of studies included barriers and facilitators about the implementation stage of the implementation process, with far fewer studies reporting on the preparation, exploration, and sustainment stages. Several studies reported across multiple stages of the implementation process.

### Barriers and facilitators reported

**Fig. 3:**
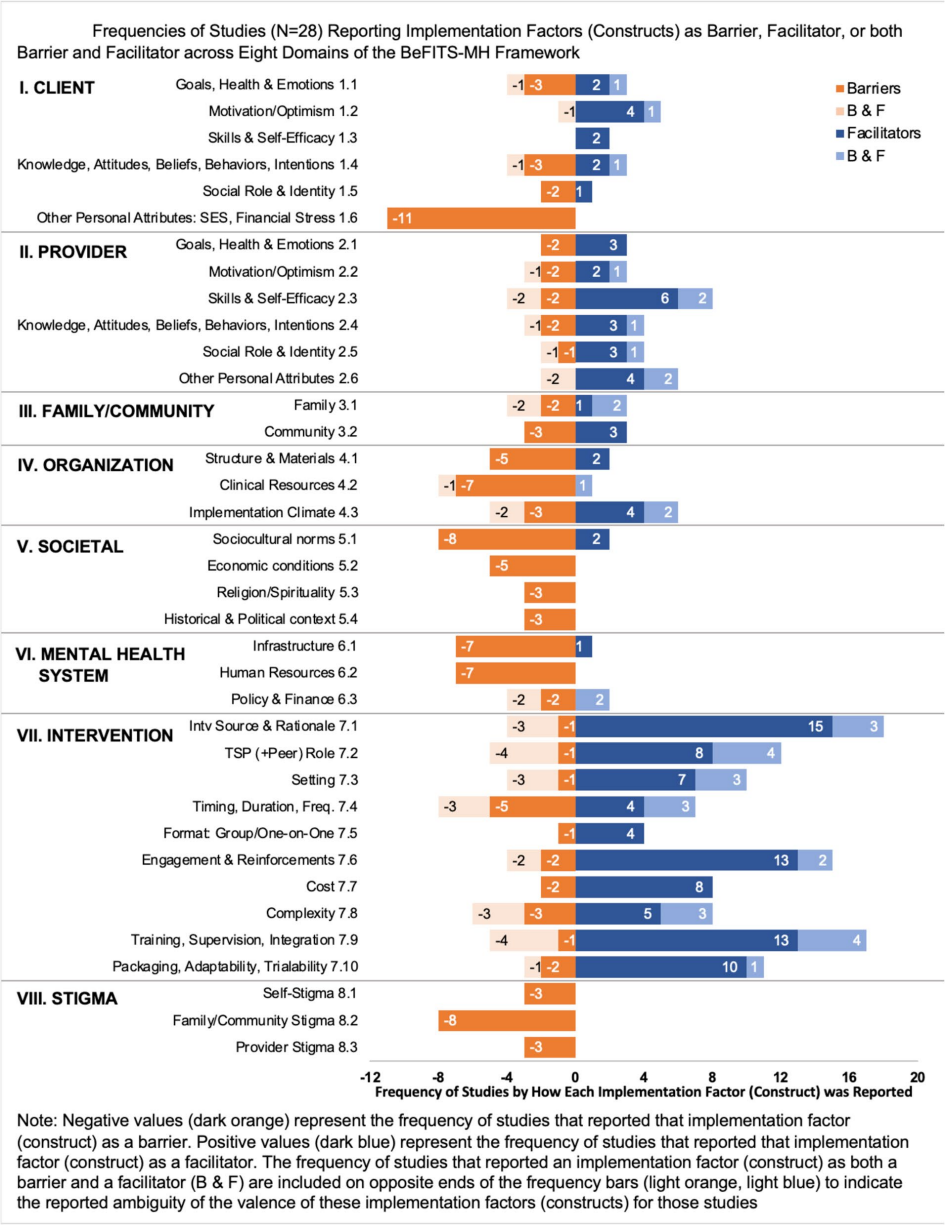
Frequencies of studies (*N* = 28) reporting implementation factors (constructs) as barrier, facilitator, or both barrier and facilitator across eight domains of the BeFITS-MH framework

Table 2 lists the 37 included articles reporting on 28 unique intervention studies, and their characteristics, including the key barriers and facilitators reported. Figure 3 presents the frequencies of each construct being reported as a barrier, facilitator, or both barrier and facilitator.

**Table 2:** Characteristics of included evidence-based task-sharing mental health intervention studies (*N* = 28 Studies from 37 articles)

Author (year)	Countr(ies)	Mental health conditions	Task-sharing model (provider and intervention type)	Implementation stage	How barriers and facilitators were assessed or derived	Key barriers reported	Key facilitators reported
***Common mental disorders (CMDs) only***							
Pacichana-Quinayáz et al. (2016) [47]	Colombia	CMDs	CHWs deliver Common Elements Treatment Approach (CETA) to Afro-Colombian victims of violence	Implementation	Assessed through in-depth interviews with providers and program administrators	Organization: Structure & materials MH system: Infrastructure Societal: Historical & political context	Client: Skills & self-efficacy Intervention: Format
Chatterjee et al. (2008) [48]	India	CMDs	Lay counselors lead collaborative stepped care intervention MANAS project (psychoeducation, antidepressants, group IPT) for CMDs in primary settings	Preparation	Assessed through interviews with providers, community members in exploration, preparation, and pilot phases	Client: Other personal attributes Intervention: Timing, duration, frequency; format	Intervention: Task-sharing provider (+peer) role; setting
*Patel et al. (2010)*^a^ [6]	India	CMDs	Lay counselors in primary care provide collaborative stepped-care intervention	Implementation	Discussed in context of study results (quantitative)	Intervention: Complexity Organization: Implementation climate	Intervention: Task-sharing provider (+peer) role Organization: Implementation climate
*Patel et al. (2011)*^a^ [49]	India	CMDs	Lay counselors in primary care provide collaborative stepped-care intervention	Implementation	Discussed in context of study results (quantitative)	N/A	Provider: Motivation/optimism
*Shinde et al. (2013)*^a^ [50]	India	CMDs	Lay counselors lead collaborative stepped care intervention MANAS project (psychoeducation, antidepressants, group interpersonal therapy) for CMDs in primary settings	Implementation	Assessed through qualitative evaluation: semi-structured interviews (SSIs) with users at two time points	Intervention: Cost (client)	Provider: Skills & self-efficacy Intervention: Task-sharing provider (+peer) role
Spagnolo et al. (2018) [51]	Tunisia	CMDs	PHCPs trained on mhGAP-based intervention to improve mental health competencies and skills	Implementation	Assessed through case study including SSIs with providers	Provider: Skills & self-efficacy; KABI Intervention: Intervention source & rationale Societal: Historical & political context	Provider: Skills & self-efficacy Intervention: Training, supervision, integration
Maulik et al. (2017) [52]	India	CMDs	CHWs identify CMDs, treated by PHCPs using mhGAP guidelines	Implementation	Assessed through mixed methods pre-post evaluation using quantitative service usage analytics	Client: KABI Intervention: Timing, duration, frequency	Intervention: Setting; Training, supervision, integration
*Tewari et al. (2017)*^a^ [53]	India	CMDs	CHWs identify CMDs, treated by PHCPs mhGAP guidelines	Implementation	Assessed through mixed methods pre-post evaluation using quantitative service usage analytics and in-depth interviews and focus group discussions with stakeholders	Client: Other personal attributes Societal: Economic conditions Stigma: Self-stigma	Client: Motivation/optimism; KABI Intervention: Task-sharing provider (+peer) role; setting
Shields et al. (2016) [54]	India	CMDs	Allopathic mental health practitioners and faith-based healers cooperate to detect and treat mental health patients via pharmacotherapy	Preparation	Assessed through mixed data: quantitative user characteristics, SSIs with users, caregivers, providers	Organization: Structure & materials MH System: Human resources Stigma: Fam/Comm stigma	Client: Motivation/optimism Intervention: Task-sharing provider (+peer) role; training, supervision, integration
Sibeko et al. (2018) [55]	South Africa	CMDs	CHWs trained on culturally adapted mhGAP program to provide chronic support including for mental illness	Preparation	Discussed in context of post-training evaluation of provider's knowledge and skills	Stigma: Provider stigma	Intervention: Engagement & reinforcements Organization: Structure & materials
Murray et al. (2014) [56]	Iraq, Thailand	CMDs (Depression, Anxiety, Traumatic stress)	Lay counselors deliver CETA	Implementation	Discussed in context of intervention development	Intervention: Complexity Organization: Structure & materials MH System: Infrastructure	Provider: Social role & identity Intervention: Engagement & reinforcement; Packaging, adaptability, trialability
Abas et al. (2016) [57]	Zimbabwe	CMDs (Depression, others)	Female CHWs deliver Problem-Solving Therapy (PST) during home visits (‘Friendship Bench’)	Sustainment	Assessed with focus group discussions and in-depth interviews with users, providers, program staff	Client: Other personal attributes Provider: Social role & identity Intervention: Training, supervision, integration	Provider: Social role & identity Intervention: Task-sharing provider (+peer) role; Setting
*Chibanda et al. (2011)*^a^ [58]	Zimbabwe	CMDs (depression, others)	Female CHWs deliver Problem-Solving Therapy (PST) during home visits (“Friendship Bench”)	Preparation	Assessed with mixed methods including questionnaire and for providers	N/A	Provider: Social role & identity Intervention: Task-sharing provider (+peer) role; setting
*Chibanda et al. (2017)*^a^ [59]	Zimbabwe	CMDs (depression, others)	Female CHWs deliver Problem-Solving Therapy (PST) during home visits (“Friendship Bench”)	Preparation	Assessed with SSIs with providers and clients post-intervention	Client: Other personal attributes Intervention: Setting	Provider: Social role & identity; skills & self-efficacy Intervention: Task-sharing provider (+peer) role
Woods-Jaeger et al. (2017) [60]	Kenya, Tanzania	CMDs (PTS, grief)	Lay counselors deliver trauma-focused Cognitive Behavioral Therapy (TF-CBT)	Sustainment	Assessed through SSIs with providers	Client: Other personal attributes Intervention: Timing, duration, frequency Fam/Comm: Community	Provider: KABI; Skills & self-efficacy Intervention: Packaging, adaptability, trialability
Dawson et al. (2016) [61]	Kenya	CMDs (PTSD, psychological distress)	CHWs deliver Problem Management Plus (PM+) for adults impacted by adversity to women in the community	Implementation	Discussed in context of intervention study results	Fam/Comm: Community	Intervention: Training, supervision, integration
O’Donnell et al. (2014) [62]	Tanzania	CMDs (PTSD)	Lay counselors deliver group-based Cognitive Behavioral Therapy (CBT) to children with symptoms of grief and/or traumatic stress	Implementation	Discussed in context of intervention study results	N/A	Provider: Other personal attributes Intervention: Training, supervision, integration
***Common mental disorders (CMDs) and comorbid conditions***							
Udedi et al. (2018) [63]	Malawi	CMDs with HIV (depression)	PHCPs, nurses, and CHWs screen and detect using algorithm-based care for depression (ABC-D) and treat with PST among patients living with HIV	Implementation	Assessed through stakeholder meetings, site visits, trainings	Provider: Skills & self-efficacy MH system: Infrastructure; human resources	Intervention: Task-sharing provider (+peer) role; engagement & reinforcements Organization: Implementation climate
***Depression only***							
Indu et al. (2018) [64]	India	Depression	PHCPs and health workers delivered psychosocial and pharmacological treatment to women with depression	Implementation	Discussed in context of intervention study results	Client: Other personal attributes Intervention: Engagement & reinforcements	Intervention: Setting; timing, duration, frequency; cost
Chowdhary et al. (2016) [65]	India	Depression	Lay counselors deliver treatment to patients with severe depression with CBT and mhGAP guidelines	Preparation; implementation	Assessed as part of intervention development, through focus group discussions with providers and in-depth interviews with supervisors and users	Intervention: Setting; timing, duration, frequency; packaging, adaptability, trialability	Provider: Other personal attributes Intervention: Intervention source & rationale; Training, supervision, integration
Adewuya et al. (2017) [66]	Nigeria	Depression	PHC workers (including doctors, nurses/midwives, community health officers, and community health extension workers) trained to detect depression among primary care patients using mhGAP guidelines	Preparation	Assessed through questionnaires administered to health workers collecting data on diagnoses and perceived challenges	Provider: KABI Organization: Clinical resources MH system: Human resources	Intervention: Intervention source & rationale Fam/Comm: Community
Petersen et al. (2012a) [11]	South Africa	Depression	CHWs deliver community-engaged mental health care	Preparation	Assessed through focus group discussions with providers and in-depth interviews with stakeholders (users, community members, mental health professionals), post-intervention	Provider: Social role & identity Societal: Sociocultural norms; historical & political context	Intervention: Format; engagement & reinforcements
*Petersen et al. (2012b)*^a^ [67]	South Africa	Depression	CHWs deliver adapted, manualized group-based Interpersonal Therapy (IPT) for female primary care patients screened with depression	Implementation	N/A	Client: Goals, health & emotions; other personal attributes	Intervention: Task-sharing provider (+peer) role; complexity; packaging, adaptability, trialability
Tomlinson et al. (2015) [68]	South Africa	Depression	CHWs provide a home visit, Cognitive Behavioral Therapy (CBT), and psychoeducation-based intervention to women with antenatal depression	Implementation	Discussed in context of study results	N/A	Intervention: Intervention source & rationale; timing, duration, frequency Organization: Implementation climate
elohilwe et al. (2019) [69]	South Africa	Depression	Lay counselors provide group CBT-based mhGAP intervention to depressed patients screened at primary care clinics	Implementation	Assessed with process evaluation consisting of in-depth interviews with stakeholders	Intervention: Engagement & reinforcements	Intervention: Task-sharing provider (+peer) role; setting; training, supervision, integration
Rahman et al. (2008) [70]	Pakistan	Depression	CHWs provide cognitive CBT-based intervention (Thinking Healthy Program) to mothers with depression	Implementation	Discussed in context of intervention development process and study results	N/A	Provider: Social role & identity Intervention: Training, supervision, integration; packaging, adaptability, trialability
Everitt-Penhale et al. (2019) [71]	South Africa	Depression	Nurses deliver an adapted CBT treatment for medication adherence and depression to individuals with HIV	Implementation	Assessed through SSIs with users post-intervention	N/A	Client: KABI Provider: Skills & self-efficacy Intervention: Task-sharing provider (+peer) role
Matsuzaka et al. (2017) [72]	Brazil	Depression	CHWs provide Interpersonal Counseling (IPC; based on IPT) to treat depression	Implementation	Discussed in context of study results	Fam/Comm: Community Societal: Religion/spirituality Stigma: Fam/Comm stigma	Provider: Goals, health & emotions; KABI Intervention: Training, supervision, integration
Munodawafa et al. (2017) [73]	South Africa	Depression: Perinatal	CHWs deliver psychosocial program (based on CBT, IPT, PST principles) for perinatal depression, part of AFFIRM in South Africa	Preparation	Assessed through SSIs with providers post-intervention	Client: Other personal attributes Provider: Skills & self-efficacy Fam/Comm: Community	Intervention: Timing, duration, frequency; cost Organization: Structure & materials
*Nyatsanza et al. (2016)*^a^ [74]	South Africa	Depression: Perinatal	CHWs deliver psychosocial program (based on CBT, IPT, PST principles) for perinatal depression, part of AFFIRM in South Africa	Exploration	N/A	Client: KABI Provider: Skills & self-efficacy Organization: Clinical Resources	Intervention: Intervention source & rationale; Training, supervision, integration; Engagement & reinforcements
Zafar et al. (2014) [75]	Pakistan	Depression: Perinatal	CHWs deliver CBT-based maternal psychosocial wellbeing intervention (Five Pillars Approach)	Implementation	Assessed through qualitative data collected in three phases (adaptation, formative, implementation) including focus group discussions and in-depth interviews with various stakeholders	Client: Other personal attributes Fam/Comm: Family Societal: Sociocultural norms	Intervention: Timing, duration, frequency; format; design quality & packaging
***Serious mental illnesses (SMIs)***							
Jordans et al. (2017) [76]	Nepal	SMI: Psychosis, epilepsy	PHCPs deliver mhGAP treatment	Implementation	Discussed in context of study results (quantitative)	N/A	Intervention: Intervention source & rationale; task-sharing provider (+peer) role; training, supervision, integration
***Serious mental illnesses (SMIs) and common mental disorders (CMDs)***							
Fils-Aimé et al. (2018) [77]	Haiti	MNS	Team including B-level psychologists, PHCPs, and CHWs treat patients with MNS disorders via mobile clinics using mhGAP guidelines and IPT	Exploration	Assessed through mixed quantitative data (quality improvement questionnaire) and qualitative (interview with implementer)	Intervention: Timing, duration, frequency Stigma: Fam/Comm stigma	Intervention: Setting; training, supervision, integration
Hanlon et al. (2014) [78]	Ethiopia, India, Nepal, South Africa, Uganda	MNS	CHWs help deliver mhGAP-informed interventions in their communities	Preparation	Assessed through qualitative ad-hoc “situation analysis tool” filled out by experts	Client: Other personal attributes Organization: Implementation climate MH System: Infrastructure	N/A
*Mendenhall et al. (2014)*^a^ [79]	Ethiopia, India, Nepal, South Africa, Uganda	MNS	CHWs help deliver mhGAP-informed interventions in their communities	Preparation	Assessed through focus group discussions and in-depth interviews with stakeholders	Provider: Skills & self-efficacy Intervention: Training, supervision, integration MH system: Infrastructure	Intervention: Intervention source & rationale; cost
Gureje et al. (2015) [80]	Nigeria	MNS: Depression, psychosis, alcohol use, epilepsy	PHCPs detect and manage MNS using the mhGAP model	Implementation	Discussed in context of post-training quantitative and qualitative data (observations)	MH System: Infrastructure; human resources Stigma: Fam/Comm stigma	Intervention: Engagement & reinforcements; packaging, adaptability, trialability; training, supervision, integration
Khoja et al. (2016) [81]	Afghanistan	MNS: Depression, psychosis, PTSD, and substance use	CHWs deliver mhGAP-based intervention to provide mental health consultation and referral to remote communities	Implementation	Discussed in context of intervention implementation and study results	N/A	Intervention: Cost; complexity Organization: Implementation climate

*CMD* Common mental disorders, *SSI* Semi-structured interview, *MNS* Mental and neural systems disorders, *MH* Mental health, *PHCPs* Primary health care providers, *CHWs* Community health workers, *KABI* Knowledge, attitude, behavior, and intentions, *Fam/Comm* Family/community

^a^Articles with Author (year) in italics refer to the same study as the last-listed non-italicized entry

When considering facilitators across the eight domains, intervention characteristics were most frequently reported, and overwhelmingly as facilitators. The six most frequently reported facilitators were all located in the intervention domain: intervention source and rationale; training, supervision, and integration; engagement and reinforcement mechanisms; packaging, adaptability, trialability; task-sharing provider’s role; and cost. The only top facilitator outside of the intervention domain was provider skills and self-efficacy. There was more variation across the domains represented by the top five barriers. Each of the five most frequently reported barriers represented five different domains: client other personal attributes (e.g., socioeconomic status, financial stress); sociocultural norms; family/community stigma; clinical resources in the organization; and mental health infrastructure and human resources.

#### Client characteristics (Domain I)

Client (other) personal attributes such as demographic factors and experiences of negative life events were identified as the most pervasive barriers in this domain. Among the demographic factors, factors tied to low socio-economic status such as poverty, unemployment, unstable housing and homelessness, and low levels of literacy or language fluency were identified as factors that hindered clients’ participation. Oftentimes, these factors limited access to interventions and the ability to fruitfully engage in programs (e.g., difficulty or an inability to travel to intervention locations [53], an unavailability to participate due to a necessity to generate income or childcare obligations [47, 48], an unwillingness to participate due to lack of monetary incentive, or an inability in understanding program materials). Clients’ negative life events caused by displacement, loss, trauma, or co-occurring adverse life situations such as living with HIV/AIDS and experiencing domestic violence were also identified as barriers to participation in task-sharing interventions. Other reported barriers included factors about clients’ knowledge about mental illness and awareness of the availability of mental health services, and clients’ goals, health, and emotions. In certain contexts, individuals did not perceive a need for or value mental health care and were therefore unaware of the potential services available or refused participation. Clients’ minimization of the extent of their problems or doubt in the efficacy of an intervention also presented barriers to widespread uptake [74].

The most salient client characteristic that facilitated implementation success was client motivation/optimism. Clients who were able to approach participation in an intervention with a readiness and confidence in success were more likely to build effective relationships with their providers, which led to better implementation and intervention outcomes. Conversely, providers faced obstacles in establishing rapport with clients whose reluctance to participate may have been motivated by constructs in higher level domains (e.g., sociocultural norms societal factors).

#### Task-sharing provider characteristics (Domain II)

Characteristics associated with the task-sharing provider were largely reported as facilitators. In contrast to clients’ other personal attributes, which were largely identified as barriers (above), providers’ other personal attributes were identified as facilitators. This is likely due to an increased acceptability of providers whose personal characteristics such as gender, age, or education align with the local contexts. In some programs, clients expressed preferences for providers who were older adult men, whereas in others, women fluent in the local language were favored. Overall, higher education generally increased acceptability due to perceived authority and mastery. Additionally, providers with higher education were perceived as being more receptive to training [55]. The most salient facilitator in this domain was provider skills and self-efficacy, which could be pre-existing as a result of prior certification or education, or developed as part of the intervention. Client’s positive perception of providers’ mastery of an intervention and itsthe associated skills and techniques, whether based on education or occupation (e.g., nurse interventionists), generally led to better intervention outcomes, with such providers possessing increased self-efficacy, more successful in establishing trust with clients, and impacting meaningful change [50, 71]. Other frequently reported facilitators in this domain included providers’ goals, health, and emotions; providers’ knowledge, attitudes, beliefs, behaviors, intentions; and providers’ social role and identity such as a provider’s links to the community or social status. Providers being skillful, having a positive attitude, believing mental illnesses are treatable conditions, and having a direct connection with their community typically nourished a strong rapport with the clients and thus led to a more successful task-sharing intervention.

#### Family and community (Domain III)

Family-related factors were sometimes identified as a barrier when family-related obligations (e.g., child care, household chores) or familial conflict hindered clients’ participation in interventions. On the other hand, providers’ relationships with family members, when successfully developed, could improve both clients’ participation in the intervention and the family’s ability to support their family member with mental illness, which contributed to better outcomes. Community factors such as community support were sometimes reported as facilitators, especially when interventions were held in small, tight-knit communities where collaboration with community members (e.g., community leaders, traditional healers) allowed for implementers to safely identify and engage potential clients, and ensure the community acceptability of the intervention [60, 67]. Successful collaborations between task-sharing providers and the community relied on the building of trust and mutual respect rooted in the understanding of sociocultural norms of the community. One example of this culturally responsive practice is the use of familial terms between clients and task-sharing providers, which helped establish older adult task-sharing providers as trusted confidants with whom community members could discuss their mental health concerns [57].

#### Organizational factors (Domain IV)

Factors operating at the organizational domain (i.e., associated with the entity implementing the task-sharing intervention) were identified primarily as barriers. Studies cited several limited clinical resources as impeding implementation. From the clients’ perspective, understaffed clinics had long waiting times and limited scheduling which reduced engagement, particularly for clients whose socioeconomic situation had constrained their availability to attend appointments. From the providers’ perspective, providers in such clinics needed to manage large caseloads which may potentially lead to burnout or decreased quality of care. Physical space constraints in health clinics also posed a major barrier, such as limiting the ease of achieving privacy for private counseling sessions with users. The absence of these resources constrained effective, sustained client participation by limiting the frequency of appointments or by making clients more reluctant to meaningfully disclose concerns or conditions due to a lack of privacy.

Implementation climate factors such as collaboration with other entities or healthcare workers on the ground in the delivery of interventions were found to be more successful. For instance, existing networks between providers facilitated referrals from task-sharing workers to specialists and other care providers [68], but a relative lack of these types of networks made implementation more difficult [78].

#### Societal factors (Domain V)

Broader, societal factors such as socioeconomic conditions, cultural norms, and historical context were identified as having a largely negative influence on intervention implementation in terms of both intervention access and ongoing engagement and delivery. Particularly, sociocultural norms emerged as the most salient barrier. Several studies emphasized the importance of accounting for cultural norms in intervention design and delivery and identified salient issues such as reluctance to discuss psychological feelings or certain taboo subjects such as death. Gender norms were also identified as factors associated with intervention delivery, as unequal gender relations limited women’s ability to seek treatment, and the gender pairings of counselors and clients often must be considered. The intervention settings’ economic conditions were identified as a challenge; communities’ levels of poverty compounded by other inequalities limited individuals’ capability to utilize services, especially among marginalized groups. Another reported barrier was religion/spirituality; beliefs in traditional healing methods were often mentioned as a deeply ingrained cultural norm that challenged client engagement in interventions. Several studies reported the historical and political context of these interventions (e.g., regime changes, wars, and histories of economic marginalization) as a barrier. For example, living in contexts with civil unrest or high rates of violence challenged clients’ capacity to prioritize mental health while concerns for safety were paramount. In some instances, clinics experienced a shortage of staffing or even closed due to the political climate. Within communities where gang violence was prevalent, clients feared for their safety traveling to and from intervention sessions and requested that their identities remain anonymous with providers [47]. Strikingly, none of the constructs in this domain emerged as major facilitators to implementation, suggesting a lack of engagement of implementers with this domain.

#### Mental health system factors (Domain VI)

Acting primarily as barriers, mental health system–level factors included infrastructure-related concerns such as lack of transportation and facilities. In one instance, a general disorganization of the mental health system failed to provide service based on need, resulting in clients with serious mental health conditions receiving inadequate care at the primary care level while a disproportionate number of clients with common mental health conditions receiving specialized care [80]. Additionally, in contexts where faith healing is commonplace, mental health systems may struggle to collaborate with preexisting systems of healing, potentially hindering the ability to reduce the mental health treatment gaps [54, 66]. Human resource issues such as the inequitable distribution of mental healthcare staffing across the country also posed as a barrier, often resulting in long wait times for clients as well as an inability to treat all clients who express a need for mental health treatment. Certain complex policy environments, such as permitting the integration of mental health care into primary care through task-sharing but providing few resources to effectively enable this, present a complicated picture of policy and finance acting both as a barrier and facilitator to these interventions. In one such context, the mental health system developed a committee to advocate for community awareness of mental illness through increased media coverage and changes in medical record keeping, ultimately reducing stigma; however, the same mental health system limited primary care providers’ involvement in pharmacological treatment, leading providers to believe only psychiatrists can prescribe such medications, despite the medications being available [51].

#### Intervention characteristics (Domain VII)

Intervention characteristics were identified in all of the studies and were overwhelmingly discussed as facilitators. Notable facilitating factors were intervention source and rationale (e.g., evidence strength and quality of the intervention; relative advantage of the intervention); high-quality training, supervision, and integration within the implementing entity, which increased provider confidence and competence; engagement and reinforcements (e.g., incorporating family and community members); packaging, adaptability, and trialability (i.e., ease of temporary adoption to test suitability); and the role of task-sharing providers. Interventions employed a variety of specific roles for task-sharing providers ranging from primary care clinicians being delegated with diagnosis, treatment, and/or referral responsibilities, to community members facilitating group-based therapy, to traditional healers and faith-based leaders being integrated into clients’ care plans. Task-sharing providers acted not only as community mobilizers for mental health but also as liaisons between the users and the rest of the intervention team. Through building partnerships with other facilities, engaging users’ families, recruiting users via community outreach, and developing community awareness towards mental health issues, task-sharing providers can strengthen community acceptability and sustainability of the intervention [80]. Other factors such as task-sharing providers’ scheduling flexibility, and client-centered and family-oriented individualized treatment also aided the service delivery process. However, some studies noted that task-sharing providers’ roles should be made more appropriate for the provider’s skillset and the community needs, and compatible with the available resources and other competing priorities. This was especially the case for peer-support workers, for whom a supportive environment is needed to forestall burnout. Several studies also highlighted that the community-based setting of some of the interventions not only facilitated access to mental health services and mitigated potential barriers such as needing transportation but also built up the intervention acceptance, increased engagement, and strengthened the rapport between implementers and the community. Barriers in the intervention domain were infrequently reported, but the timing, frequency, and duration—particularly that the interventions were too short—and complexity were the most frequently noted challenges. Intervention cost acted as both a facilitator when free or low cost, and as a barrier when inaccessible financially. This is to be expected as many of the included studies were conducted in communities where poverty is prevalent.

#### Stigma (Domain VIII)

Mental illness stigma, which also operates across different levels like the intervention domain, was solely reported as hindering implementation success and when present, hindered implementation substantially. Self-stigma by individuals with mental illness was found to be a barrier to seeking psychological/psychiatric treatment and was likely directly linked to high drop-out rates. Enacted stigma toward mental illness (i.e., clients’ experiences of discrimination from family and community members) also discouraged treatment-seeking and intervention access. In some instances, official diagnoses of mental health illness resulted in decreased community perceptions of trustworthiness [51]. Therefore, an intervention’s ability to maintain patient confidentiality was paramount to the client’s acceptance and adherence to programs. Stigma in the healthcare setting was found to be a barrier when stigmatizing attitudes were found to be held or expressed by healthcare and task-sharing providers. Such providers viewed clients as potentially dangerous to themselves and others, hampering effective implementation [55]. Stigma related to mental health was also reported as being embedded within mental health systems at the structural level and was suggested to be related to a systemic lack of training and education about the treatment of mental illness at the primary care level. Some studies reported that the task-sharing strategy reduced mental health stigma, but, as expected, no study reported stigma as enhancing either implementation or intervention outcomes.

## Discussion

In this review, we used implementation science frameworks to develop a multi-dimensional conceptual model for the barriers and facilitators of mental health task–sharing interventions (i.e., the BeFITS-MH framework) and used it to classify the barriers and facilitators reported in the included studies. This process showed that the most prevalent facilitators occur in domains that are more amenable to adaptation (i.e., the provider and intervention domains), while salient barriers occur in domains that are challenging to modulate or intervene upon—these include constructs in the client characteristics as well as the broader societal and structural levels of influence (i.e., the organizational, mental health system domains). Other notable trends include constructs in the family and community domains occurring as barriers and as facilitators roughly equally, and stigma constructs acting exclusively as barriers. These findings have important implications for ongoing and future implementation of evidence-based task-sharing mental health intervention models, including the promise of leveraging task-sharing intervention characteristics as sites of continued innovation; the importance of but relative lack of engagement with constructs in macro-level domains (e.g. organizational characteristics, stigma); and the need for more delineation of strategies for task-sharing mental health interventions that researchers and implementers can employ to enhance implementation within and across levels.

That task-sharing intervention factors were the most reported constructs and mostly acted as facilitators is expected, as intervention factors are more amenable to change compared to structural or personal factors. In particular, defining elements of task-sharing (e.g., the types of task-sharing provider roles and the training and supervision models) have been specifically designed to be inherently beneficial and are demonstrated to be a strength of this approach [4]. Given the wide range of task-sharing provider roles employed and the salience of provider characteristics as facilitators, it is important to ensure that the roles of the task-sharing providers are appropriate for the providers’ knowledge, attitude, skills, and confidence, as well as the clients’ needs. One way of ensuring that provider roles are congruent with both their context and their competencies is ensuring high-quality training and consistent supervision [80]. The WHO has long recognized training and supervision as a crucial element of a successful task-sharing intervention [82, 83], and efforts to enable intervention sites to design and monitor their training and supervision in a systematic, locally specific, and pragmatic way are needed and ongoing [84–86]. These efforts include tools such as ENACT, which can be used to monitor therapist competence [85], and recent initiatives such as the WHO’s EQUIP: Ensuring Quality in Psychosocial Support, which seeks to develop and compile comprehensive sets of quality resources for psychological and psychosocial support interventions globally [87, 88].

The fit of task-sharing providers’ roles can also heavily depend on a key facilitator noted in another domain: the task-sharing provider’s personal characteristics, such as their age, gender, and educational background. Alignment of these provider characteristics with the community in which they work can improve the acceptability of task-sharing interventions [28, 89, 90]. Interventions s are often enhanced by having task-sharing providers with strong existing community ties and credibility, shared lived experience, and non-stigmatizing attitudes [91, 92]. In particular, the involvement of individuals with similar lived experience as the clients (e.g., peer support services) is increasingly recognized as a potentially powerful task-sharing strategy to both increase the acceptability of the intervention for clients and help them navigate and resist sources and drivers of stigma [92, 93]. Service user involvement is thus a strategy that should be further studied.

This review also underscored that there are a variety of factors that present as major barriers to the implementation of task-sharing mental health interventions at and across levels, including stigma. Trust between all actors (primary care providers, task-sharing providers, families, and people with mental health concerns) must be established to promote treatment initiation and maintain engagement [94], in part by countering the prevailing stigmatizing attitudes and perceptions [55, 92]. This is necessarily a difficult endeavor, as stigma operates across and between many stakeholders and levels. However, in addition to internalized, public, and structural stigma toward mental health conditions generally, task-sharing providers themselves may be stigmatized by their colleagues—i.e., other professionals on the healthcare team [25]. Yet, little is currently known about how to mitigate these stigmatizing attitudes, especially those relating to peer providers’ “liminal positions” as both service users and providers (see [95]). Additionally, multi-level stigma interventions are rare [96], and this review suggests that they are both necessary to mitigate stigma as a barrier to task-sharing mental health interventions and may have to incorporate specific components that are responsive to the unique characteristics, barriers, and facilitators of the task-sharing intervention model.

In addition to the importance of developing and applying new and existing implementation strategies to enhance known facilitators (e.g., provider roles, training, and supervision), another key takeaway from this review is the need to develop and adopt innovative strategies for addressing macro-level barriers. This need for task-sharing interventions to be more responsive to and resistant against macro-level factors is echoed by emerging research that has highlighted the role that structural forces play in not only impeding implementation in their own right but also perpetuating and reinforcing other barriers (e.g., stigma, deprioritization of mental health care) [97–99]. In LMICs, as in much of the world, mental health is consistently relegated to a secondary or even tertiary concern [1, 100, 101], and governmental-level forces and policies that perpetuate this lack of parity have exerted pervasive negative impact on resources allotted to mental healthcare and the individual lives of people who deserve it [102]. In terms of economic impact, the inadequacy of these policies in addressing the mental health gap is projected to cost the global economy a staggering $6 trillion US dollars by 2030 [5, 103]. More importantly, mental health resource deficiencies have a cascading effect on individuals and communities with intersecting vulnerabilities (e.g., individuals affected by communicable and non-communicable diseases, communities affected by humanitarian crises) by exacerbating and compounding the human rights violations, discrimination, and stigma they often experience [5]. Thus, addressing the mental health gap requires a strengthening of the mental healthcare delivery system, including via task-sharing mental health interventions and the implementation strategies typically included therein.

However, the science of developing implementation strategies that could be used to address barriers or to leverage facilitators of task-sharing mental health interventions across all levels is only in its nascent stages. Despite the frequent recognition of the many barriers and facilitators to evidence-based task-sharing interventions, this review found that few studies clearly specified implementation strategies [also see 18]. Although recent implementation research has contributed to the development of guidelines for the selection and tailoring [104] as well as a comprehensive classification [105] of implementation strategies, this is rarely done [106]. The systematic, comprehensive synthesis of barriers and facilitators presented in this review hopefully will propel the field forward by helping to guide the selection and reporting of specific implementation strategies that can improve the implementation and intervention outcomes for task-sharing mental health interventions. For example, knowing that training, supervision, and integration is a highly salient facilitator could help practitioners and researchers consider how related strategies such as “conduct ongoing training,” “facilitation,” “make training dynamic,” and “provide clinical supervision” could be applicable to their intervention.

Further, this review suggests that future implementation strategies for task-sharing developed in LMICs should seek to address prevalent and pervasive meso- and macro-level barriers and, whenever possible, prioritize aligning with organizational and mental health system efforts to leverage any existing or emerging meso- and macro-level facilitators (e.g., “systems considerations,” [107, 108]). Additionally, linking this review’s findings on the implementation determinants of the task-sharing approach to the current knowledge base on implementation strategies can contribute to both the further specification of existing strategies and perhaps the development of new and distinct implementation strategies. These new and distinct implementation strategies could prove applicable to task-sharing interventions for conditions other than mental health, to task-sharing interventions in settings other than LMICs (e.g., high-income countries [109]), and potentially to other intervention models besides task-sharing.

### Limitations

Several limitations should be considered. First, the articles included in this review were peer-reviewed journal articles, meaning the grey literature was not utilized. While limiting our criteria to peer-reviewed articles ensures a baseline study quality and rigor, it also makes the studies included in this review subject to publication bias, wherein studies finding statistically significant results or coming from higher-resourced study teams have a higher likelihood of ultimately being identified for inclusion. Published studies were probably more likely to report on positive intervention characteristics that contributed to the significant results achieved by the study, which might have resulted in an overreporting of intervention characteristics as facilitators. Additionally, all studies included for review were in the English language; this criterion was necessary given our team’s linguistic limitations, but limits our access to studies published in local-language journals which may have been a robust source of additional context-rich research given our global focus. Lastly, inherent to the systematic review study design is the reliance on the post-hoc analyses of the included studies; the findings are subject to the methodological rigor and biases of the primary studies. Although all included articles passed their respective quality assessment criteria, barriers and facilitators may be especially likely to lack rigorous reporting or be absent from results entirely given the lack of measurement tools. Thus, in an attempt to mitigate this concern and be properly inclusive, we also critically appraised the discussion sections of each article to allow inclusion of data-grounded discussion of barriers and facilitators .

## Conclusion

Task-sharing in mental health services continues to be adopted widely, especially to alleviate the mental health treatment gap in many LMICs. Our results underscore a need for greater focus on under-researched geographic areas (e.g., Latin America), populations (e.g., people with severe mental illness), and EBPs. Furthermore, we suggest that future studies examine barriers and facilitators and develop implementation strategies across all phases of implementation—especially in the sustainment phase, which will be essential to understand as task-sharing continues to proliferate and become embedded in mental health systems globally [106].

By applying our multi-dimensional BeFITS-MH conceptual framework, which integrated domains and constructs specified in prominent implementation science frameworks, this review offers a comprehensive analysis of barriers and facilitators identified in evidence-based task-sharing mental health interventions conducted in LMICs. Results point to the need for immediate remedies to address material deficiencies in the mental healthcare system and community clinics as a whole, in addition to more complex solutions to mitigate the detrimental effects of mental health stigma and to navigate sociocultural norms in the context of treatment. Barriers and facilitators (also, “determinants”) are often situated as key factors that lead to implementation outcomes [14, 35]. This review serves as an important step in explicating this relationship by improving the systematic identification of implementation determinants via the BeFITS-MH conceptual model so that practitioners and researchers can act on and modify these constructs while knowing how they commonly operate and interact with other constructs across different domains. This can better inform implementers and researchers in selecting and tailoring specific implementation strategies that can improve implementation and intervention outcomes.

Furthermore, the lack of valid and pragmatic assessments for implementation-related factors and outcomes has been noted in the health services and implementation science literature [110, 111] and has been echoed by researchers across health-related interventions [112]. However, current efforts to address these challenges (e.g., [113, 114]) remain largely focused on compiling existing measures. The framework presented in this review and the results are informing ongoing work from our team in the development of a valid, comprehensive, and pragmatic tool to assess barriers and facilitators of task-sharing mental health interventions in LMIC settings [115]. This tool will support measurement of implementation determinants in the global context and serve to advance our understanding of and responsiveness to the factors that contribute to the successful implementation of task-sharing mental health interventions in order to reduce the prominent mental health treatment gap in low-resource settings.

## Supplementary Information

**Additional file 1: A.** Barriers and facilitators to implementation of evidence-based task-sharing mental health interventions in low- and middle-income countries: A systematic review using implementation science frameworks: PRISMA Checklist. **B.** Barriers and facilitators to implementation of evidence-based task-sharing mental health interventions in low- and middle-income countries: A systematic review using implementation science frameworks: Search Syntax. **C.** Barriers and facilitators to implementation of evidence-based task-sharing mental health interventions in low- and middle-income countries: A systematic review using implementation science frameworks: Quality Assessment Information by Study Type.

## Data Availability

Data sharing is not applicable to this article as no datasets were generated or analyzed during the current study.

## Abbreviations

BeFITS-MH: Barriers and Facilitators in Implementation of Task-Sharing Mental Health
CAPs: Complementary and alternative providers
CBT: Cognitive Behavioral Therapy
CETA: Common Elements Treatment Approach
CFIR: Consolidated Framework for Implementation Research
CHWs: Community Health Workers
CMDs: Common mental disorders
EBP: Evidence-based practice
IPT: Interpersonal therapy
LMICs: Low- and middle-income countries
mhGAP: Mental Health Gap Action Programme
PHCPs: Primary health care providers
PRISMA: Preferred Reporting Items for Systematic Reviews and Meta-Analyses
PM+: Problem Management Plus
PST: Problem-Solving Therapy
SMI: Serious mental illness
TDF: Theoretical Domains Framework
WHO: World Health Organization

## Footnotes

1 The 14 domains of the Theoretical Domains Framework are (1) knowledge; (2) skills; (3) social/ professional role and identity; (4) beliefs about capabilities; (5) optimism; (6) beliefs about consequences; (7) reinforcement; (8) intention; (9) goals; (10) memory, attention, and decision processes; (11) environmental context and resources; (12) social influences; (13) emotion; and (14) behavioral regulation.
