# Supplementary material for: Barriers and facilitators to implementation of evidence-based task-sharing mental health interventions in low- and middle-income countries: a systematic review using implementation science frameworks
Source: Implement Sci. 2022 Jan 12;17:4. doi: 10.1186/s13012-021-01179-z (PMC8756725; doi:10.1186/s13012-021-01179-z)
Supplement: Supplementary file 1 — Additional file 1: A. Barriers and facilitators to implementation of evidence-based task-sharing mental health interventions in low- and middle-income countries: A systematic review using implementation science frameworks: PRISMA Checklist. B. Barriers and facilitators to implementation of evidence-based task-sharing mental health interventions in low- and middle-income countries: A systematic review using implementation science frameworks: Search Syntax. C. Barriers and facilitators to implementation of evidence-based task-sharing mental health interventions in low- and middle-income countries: A systematic review using implementation science frameworks: Quality Assessment Information by Study Type. [file 13012_2021_1179_MOESM1_ESM.zip › EBP_TSMH SysRev AddFile 1B Search SyntaxR4.docx]

# Additional File 1B for *Barriers and facilitators to implementation of evidence-based task-sharing mental health interventions in low- and middle-income countries: A systematic review using implementation science frameworks*: Search Syntax

| Database | Syntax |
| --- | --- |
| PubMed | (("Developing Countries"[Mesh]  OR  (("emerging"[tiab] OR "developing"[tiab] OR "less developed"[tiab] OR "lesser developed"[tiab] OR "under developed"[tiab] OR "underdeveloped"[tiab] OR "under-developed"[tiab] OR "low income"[tiab] OR "low-income"[tiab] OR "lower income"[tiab] OR "lower-income"[tiab] OR "middle income"[tiab] OR "middle-income"[tiab] OR "underserved"[tiab] OR "under-served"[tiab] OR "under served"[tiab] OR "poor"[tiab] OR "poorer"[tiab] OR "poorest"[tiab] OR "deprived"[tiab] OR "transitional"[tiab]) **AND** ("country"[tiab] OR "countries"[tiab] OR "nation"[tiab] OR "nations"[tiab] OR "population"[tiab] OR "populations"[tiab] OR "economy"[tiab] OR "economies"[tiab]) OR "world"[tiab]))  OR  "low gdp"[tiab] OR "low gnp"[tiab] OR "low gross domestic"[tiab] OR "low gross national"[tiab] OR "lower gdp"[tiab] OR "lower gross domestic"[tiab] OR LMIC[tiab] OR LMICs[tiab] OR "lami country"[tiab] OR "lami countries"[tiab] OR "third world"[tiab]  OR  Africa[tiab] OR "africa"[MeSH Terms:noexp] OR "Africa, Northern"[Mesh:noexp] OR "Africa, Central"[Mesh:noexp] OR "Africa, Eastern"[Mesh:noexp] OR "Africa, Southern"[Mesh:noexp] OR "Africa, Western"[Mesh:noexp] OR Asia[tiab] OR "asia"[MeSH Terms:noexp] OR "Asia, Central"[Mesh:noexp] OR "Asia, Southeastern"[Mesh:noexp] OR "Asia, Western"[Mesh:noexp] OR Caribbean[tiab] OR "Caribbean Region"[Mesh:noexp] OR "West Indies"[tiab] OR "West Indies"[Mesh:noexp] OR "South America"[tiab] OR "Latin America"[tiab] OR "Central America"[tiab] OR "South America"[Mesh:noexp] OR "Latin America"[Mesh:noexp] OR "Central America"[Mesh:noexp] OR "Atlantic Islands"[tiab] OR "Atlantic Islands"[Mesh:noexp] OR "Commonwealth of Independent States"[tiab] OR "Commonwealth of Independent States"[Mesh:noexp] OR "Pacific Islands"[tiab] OR "Pacific Islands"[Mesh:noexp] OR "Indian Ocean Islands"[tiab] OR "Indian Ocean Islands"[Mesh:noexp] OR "Eastern Europe"[tiab] OR "Europe, Eastern"[Mesh:noexp] OR "Southern African Development Community"[all fields] OR "East African Community"[all fields] OR "West African Health Organisation"[all fields] OR "West African Health Organization"[all fields] OR "Pan American Health Organization"[all fields] OR "Pan American Health Organisation"[all fields] OR ("pan american health organization"[MeSH Terms] OR  ("pan"[All Fields] AND "american"[All Fields] AND "health"[All Fields] AND "organization"[All Fields]) OR  "pan american health organization"[All Fields] OR "paho"[All Fields])  OR  Afghanistan[tiab] OR Albania[tiab] OR Algeria[tiab] OR Angola[tiab] OR Armenia[tiab] OR Armenian[tiab] OR Azerbaijan[tiab] OR Bangladesh[tiab] OR Benin[tiab] OR Byelarus[tiab] OR Byelorussian[tiab] OR Belarus[tiab] OR Belorussian[tiab] OR Belorussia[tiab] OR Belize[tiab] OR Bhutan[tiab] OR Bolivia[tiab] OR Bosnia[tiab] OR Herzegovina[tiab] OR Hercegovina[tiab] OR Bosnia-Herzegovina[tiab] OR Botswana[tiab] OR Brasil[tiab] OR Brazil[tiab] OR Bulgaria[tiab] OR "Burkina Faso"[tiab] OR "Burkina Fasso"[tiab] OR "Upper Volta"[tiab] OR Burundi[tiab] OR Urundi[tiab] OR Cambodia[tiab] OR "Khmer Republic"[tiab] OR "Kampuchea"[tiab] OR Cameroon[tiab] OR Cameroons[tiab] OR Cameron[tiab] OR "Cape Verde"[tiab] OR "Central African Republic"[tiab] OR Chad[tiab] OR China[tiab] OR Colombia[tiab] OR Comoros[tiab] OR "Comoro Islands"[tiab] OR Comores[tiab] OR Mayotte[tiab] OR Congo[tiab] OR Zaire[tiab] OR "Costa Rica"[tiab] OR "Cote d'Ivoire"[tiab] OR "Ivory Coast"[tiab] OR Cuba[tiab] OR Czechoslovakia[tiab] OR Slovakia[tiab] OR Djibouti[tiab] OR "French Somaliland"[tiab] OR Dominica[tiab] OR "Dominican Republic"[tiab] OR "East Timor"[tiab] OR "Timor Leste"[tiab] OR Ecuador[tiab] OR Egypt[tiab] OR "El Salvador"[tiab] OR Eritrea[tiab] OR Ethiopia[tiab] OR Fiji[tiab] OR Gabon[tiab] OR "Gabonese Republic"[tiab] OR Gambia[tiab] OR Gaza[tiab] OR "Georgia Republic"[tiab] OR "Georgian Republic"[tiab] OR Ghana[tiab] OR "Gold Coast"[tiab] OR Grenada[tiab] OR Guatemala[tiab] OR Guinea[tiab] OR Guiana[tiab] OR Guyana[tiab] OR Haiti[tiab] OR Honduras[tiab] OR India[tiab] OR Maldives[tiab] OR Indonesia[tiab] OR Iran[tiab] OR Iraq[tiab] OR Jamaica[tiab] OR Jordan[tiab] OR Kazakhstan[tiab] OR Kazakh[tiab] OR Kenya[tiab] OR Kiribati[tiab] OR Korea[tiab] OR Kosovo[tiab] OR Kyrgyzstan[tiab] OR Kirghizia[tiab] OR "Kyrgyz Republic"[tiab] OR Kirghiz[tiab] OR Kirgizstan[tiab] OR "Lao PDR"[tiab] OR Laos[tiab] OR Lebanon[tiab] OR Lesotho[tiab] OR Basutoland[tiab] OR Liberia[tiab] OR Libya[tiab] OR Macedonia[tiab] OR Madagascar[tiab] OR "Malagasy Republic"[tiab] OR Malaysia[tiab] OR Malaya[tiab] OR Malay[tiab] OR Sabah[tiab] OR Sarawak[tiab] OR Malawi[tiab] OR Nyasaland[tiab] OR Mali[tiab] OR "Marshall Islands"[tiab] OR Mauritania[tiab] OR Mauritius[tiab] OR "Agalega Islands"[tiab] OR Melanesia[tiab] OR Mexico[tiab] OR Micronesia[tiab] OR "Middle East"[tiab] OR Moldova[tiab] OR Moldovia[tiab] OR Moldovian[tiab] OR Mongolia[tiab] OR Montenegro[tiab] OR Morocco[tiab] OR Ifni[tiab] OR Mozambique[tiab] OR Myanmar[tiab] OR Myanma[tiab] OR Burma[tiab] OR Namibia[tiab] OR Nepal[tiab] OR Nicaragua[tiab] OR Niger[tiab] OR Nigeria[tiab] OR Muscat[tiab] OR Pakistan[tiab] OR Palau[tiab] OR Palestine[tiab] OR Panama[tiab] OR Paraguay[tiab] OR Peru[tiab] OR Philippines[tiab] OR Philipines[tiab] OR Phillipines[tiab] OR Phillippines[tiab] OR Romania[tiab] OR Rumania[tiab] OR Roumania[tiab] OR Rwanda[tiab] OR Ruanda[tiab] OR "Saint Kitts"[tiab] OR "St Kitts"[tiab] OR Nevis[tiab] OR "Saint Lucia"[tiab] OR "St Lucia"[tiab] OR "Saint Vincent"[tiab] OR "St Vincent"[tiab] OR Grenadines[tiab] OR Samoa[tiab] OR Samoan Islands[tiab] OR "Sao Tome"[tiab] OR Senegal[tiab] OR Serbia[tiab] OR Montenegro[tiab] OR "Sierra Leone"[tiab] OR "Sri Lanka"[tiab] OR Ceylon[tiab] OR "Solomon Islands"[tiab] OR Somalia[tiab] OR Sudan[tiab] OR Suriname[tiab] OR Surinam[tiab] OR Swaziland[tiab] OR Syria[tiab] OR Syrian[tiab] OR Tajikistan[tiab] OR Tadzhikistan[tiab] OR Tadjikistan[tiab] OR Tadzhik[tiab] OR Tanzania[tiab] OR Thailand[tiab] OR Togo[tiab] OR Togolese Republic[tiab] OR Tonga[tiab] OR Tunisia[tiab] OR Turkey[tiab] OR Turkmenistan[tiab] OR Turkmen[tiab] OR Tuvalu[tiab] OR Uganda[tiab] OR Ukraine[tiab] OR Uzbekistan[tiab] OR Uzbek[All Fields] OR Vanuatu[tiab] OR New Hebrides[tiab] OR Vietnam[tiab] OR "Viet Nam"[tiab] OR "Viet-Nam"[tiab] OR "West Bank"[tiab] OR Yemen[tiab] OR Yugoslavia[tiab] OR Zambia[tiab] OR Zimbabwe[tiab] OR Rhodesia[tiab] OR "afghanistan"[MeSH Terms]  OR  "albania"[MeSH Terms] OR "algeria"[MeSH Terms] OR "American Samoa"[Mesh] OR "angola"[MeSH Terms] OR "armenia"[MeSH Terms] OR "azerbaijan"[MeSH Terms] OR "Baltic States"[Mesh] OR "bangladesh"[MeSH Terms] OR "benin"[MeSH Terms] OR "Republic of Belarus"[Mesh] OR "belize"[MeSH Terms] OR "bhutan"[MeSH Terms] OR "bolivia"[MeSH Terms] OR "botswana"[MeSH Terms] OR "brazil"[MeSH Terms] OR "bulgaria"[MeSH Terms] OR "Burkina Faso"[Mesh] OR "burundi"[MeSH Terms] OR "cambodia"[MeSH Terms] OR "cameroon"[MeSH Terms] OR "Central African Republic"[Mesh] OR "chad"[MeSH Terms] OR "china"[MeSH Terms] OR "colombia"[MeSH Terms] OR "comoros"[MeSH Terms] OR "congo"[MeSH Terms] OR "Costa Rica"[Mesh] OR "Cote d'Ivoire"[Mesh] OR "cuba"[MeSH Terms] OR "czechoslovakia"[MeSH Terms] OR "slovakia"[MeSH Terms] OR "djibouti"[MeSH Terms] OR "Democratic Republic of the Congo"[Mesh] OR "Democratic People's Republic of Korea"[Mesh] OR "dominica"[MeSH Terms] OR "Dominican Republic"[Mesh] OR "ecuador"[MeSH Terms] OR "egypt"[MeSH Terms] OR "El Salvador"[Mesh] OR "eritrea"[MeSH Terms] OR "ethiopia"[MeSH Terms] OR "fiji"[MeSH Terms] OR "French Guiana"[Mesh] OR "gabon"[MeSH Terms] OR "gambia"[MeSH Terms] OR "Georgia (Republic)"[Mesh] OR "ghana"[MeSH Terms] OR "grenada"[MeSH Terms] OR "guatemala"[MeSH Terms] OR "guinea"[MeSH Terms] OR "Guinea-Bissau"[Mesh] OR "guyana"[MeSH Terms] OR "haiti"[MeSH Terms] OR "honduras"[MeSH Terms] OR "Independent State of Samoa"[Mesh] OR "india"[MeSH Terms] OR "indonesia"[MeSH Terms] OR "iran"[MeSH Terms] OR "iraq"[MeSH Terms] OR "jamaica"[MeSH Terms] OR "jordan"[MeSH Terms] OR "kazakhstan"[MeSH Terms] OR "kenya"[MeSH Terms] OR "korea"[MeSH Terms] OR "kyrgyzstan"[MeSH Terms] OR "laos"[MeSH Terms] OR "lebanon"[MeSH Terms] OR "lesotho"[MeSH Terms] OR "liberia"[MeSH Terms] OR "libya"[MeSH Terms] OR "Macedonia (Republic)"[Mesh] OR "madagascar"[MeSH Terms] OR "malawi"[MeSH Terms] OR "malaysia"[MeSH Terms] OR "mali"[MeSH Terms] OR "mauritania"[MeSH Terms] OR "mauritius"[MeSH Terms] OR "Melanesia"[Mesh] OR "mexico"[MeSH Terms] OR "micronesia"[MeSH Terms] OR "Middle East"[Mesh:noexp] OR "moldova"[MeSH Terms] OR "mongolia"[MeSH Terms] OR "montenegro"[MeSH Terms] OR "morocco"[MeSH Terms] OR "mozambique"[MeSH Terms] OR "myanmar"[MeSH Terms] OR "namibia"[MeSH Terms] OR "nepal"[MeSH Terms] OR "nicaragua"[MeSH Terms] OR "niger"[MeSH Terms] OR "nigeria"[MeSH Terms] OR "pakistan"[MeSH Terms] OR "palau"[MeSH Terms] OR "panama"[MeSH Terms] OR "Papua New Guinea"[Mesh] OR "paraguay"[MeSH Terms] OR "peru"[MeSH Terms] OR "philippines"[MeSH Terms] OR "Republic of Korea"[Mesh] OR "romania"[MeSH Terms] OR "rwanda"[MeSH Terms] OR "Saint Lucia"[Mesh] OR "Saint Vincent and the Grenadines"[Mesh] OR "samoa"[MeSH Terms] OR "senegal"[MeSH Terms] OR "serbia"[MeSH Terms] OR "montenegro"[MeSH Terms] OR "sierra leone"[MeSH Terms] OR "Sri Lanka"[Mesh] OR "somalia"[MeSH Terms] OR "south africa"[MeSH Terms] OR "sudan"[MeSH Terms] OR "suriname"[MeSH Terms] OR "swaziland"[MeSH Terms] OR "syria"[MeSH Terms] OR "tajikistan"[MeSH Terms] OR "tanzania"[MeSH Terms] OR "thailand"[MeSH Terms] OR "togo"[MeSH Terms] OR "tonga"[MeSH Terms] OR "tunisia"[MeSH Terms] OR "turkey"[MeSH Terms] OR "turkmenistan"[MeSH Terms] OR "uganda"[MeSH Terms] OR "ukraine"[MeSH Terms] OR "uzbekistan"[MeSH Terms] OR "vanuatu"[MeSH Terms] OR "vietnam"[MeSH Terms] OR "yemen"[MeSH Terms] OR "yugoslavia"[MeSH Terms] OR "zambia"[MeSH Terms] OR "zimbabwe"[MeSH Terms])  AND  ("Community Health Workers"[MeSH] OR "Allied Health Personnel"[MeSH] OR "Home Health Aides"[MeSH] OR "Home Health Aides"[MeSH] OR "Nursing Assistants"[MeSH] OR "Psychiatric Aides"[MeSH] OR "Caregivers"[MeSH] OR "Hospital Volunteers"[MeSH]  OR  (("community"[tiab] OR "lay"[tiab] OR "village"[tiab] OR "home"[tiab] OR "frontline"[tiab] OR "front-line"[tiab] OR "mental"[tiab] OR "nonphysician"[tiab] OR "untrained"[tiab] OR "trained"[tiab] OR "voluntary"[tiab] OR "nonmedical"[tiab] OR "non-medical"[tiab] OR "nonspecialist"[tiab] OR "non-specialist"[tiab] OR "nonprofessional"[tiab] OR "non-professional"[tiab] OR "paraprofessional"[tiab] OR "para-professional"[tiab] OR "extension"[tiab] OR "auxiliary"[tiab]) **AND** ("worker"[tiab] OR "workers"[tiab] OR "practitioners"[tiab] OR "representatives"[tiab] OR "advisors"[tiab] OR "counselors"[tiab] OR "health aides"[tiab] OR "guides"[tiab] OR "attendants"[tiab] OR "care givers"[tiab] OR "caregivers"[tiab] OR "consultants"[tiab] OR "volunteers"[tiab] OR "extenders"[tiab]))  OR  "promotora"[tiab] OR "promotores"[tiab] OR ("patient"[tiab] **AND** ("navigators"[tiab] OR "navigator"[tiab] OR "navigation"[tiab]))  OR "barefoot doctors"[tiab] OR "barefoot doctor"[tiab] OR (("task"[tiab]  OR  "tasks"[tiab]) AND ("shift"[tiab] OR "shifted"[tiab] OR "shifts"[tiab] OR "shifting"[tiab] OR "share"[tiab] OR "shared"[tiab] OR "sharing"[tiab] OR "delegation"[tiab] OR "delegating"[tiab] OR "substitution"[tiab] OR "substituting"[tiab])) OR (("shortage"[tiab] OR "substitution"[tiab] OR "substituted"[tiab] OR "substitute"[tiab] OR "substituting"[tiab] OR "substitutes"[tiab] OR "delegate"[tiab] OR "delegating"[tiab] OR "delegates"[tiab] OR "delegation"[tiab] OR "delegated"[tiab]) **AND** ("Physicians"[MeSH] OR "Health Personnel"[MeSH] OR "Nurses"[MeSH] OR "Nurses, Community Health"[MeSH] OR "Nurses, International"[MeSH] OR "Nurses, Public Health"[MeSH] OR "Personnel, Hospital"[MeSH] OR "Hospital Volunteers"[MeSH] OR "Medical Staff, Hospital"[MeSH] OR "Nursing Staff, Hospital"[MeSH] OR "physicians"[tiab] OR "doctors"[tiab] OR "trained personnel"[tiab] OR "health workforce"[tiab] OR "health care workforce"[tiab] OR "healthcare workforce"[tiab] OR "health workers"[tiab] OR "health care workers"[tiab] OR "healthcare workers"[tiab] OR "health care providers"[tiab] OR "health providers"[tiab] OR "healthcare providers"[tiab] OR "health professionals"[tiab] OR "healthcare professionals"[tiab] OR "health care professionals"[tiab] OR "health practitioners"[tiab] OR "nurses"[tiab] OR "psychologists"[tiab] OR "psychiatrists"[tiab] OR "clinicians"[tiab]))  OR  ("nurse led"[All Fields] OR "primary health care nurse"[All Fields] OR "primary health care nurses"[All Fields] OR "primary health care nursing"[All Fields])  OR ("role"[All Fields] **AND** ("nurse"[All Fields] OR "nurses"[All Fields] OR "nursing"[All Fields]))  OR "community based"[tiab] OR ("community"[tiab] AND "intervention"[tiab]) **AND** "self help group"[tiab] OR "self-help groups"[tiab] OR "support groups"[tiab] OR (("social"[tiab] OR "psychosocial"[tiab]) **AND** ("care"[tiab] OR "support"[tiab])))  AND  ("Mental Health"[MeSH] OR "Mental Health Services"[MeSH] OR "Community Mental Health Services"[MeSH] OR "Community Mental Health Centers"[MeSH] OR "Mental Health Recovery"[MeSH] OR "Psychiatric Nursing"[MeSH] OR "Psychiatric Rehabilitation"[MeSH] OR "Psychology"[MeSH] OR "Resilience, Psychological"[MeSH] OR (("mental"[tiab] OR "psychological"[tiab] OR "psychosocial"[tiab] OR "psycho-social"[tiab] OR  "emotional"[tiab]) AND ("health"[tiab] OR  "wellbeing"[tiab] OR  "well-being"[tiab] OR "well being"[tiab] OR "resilience"[tiab] OR "resiliency"[tiab] OR "symptoms"[tiab])) OR (("mental"[tiab] OR "community"[tiab]) AND ("health service"[tiab] OR "health services"[tiab] OR "health center"[tiab] OR "health centers"[tiab] OR "health centre"[tiab] OR "health centres"[tiab])) OR (("mental health"[tiab] OR "psychiatric"[tiab]) AND ("recovery"[tiab] OR "nursing"[tiab] OR "rehabilitation"[tiab])) OR "Mental Disorders"[MeSH] OR "Anxiety Disorders"[MeSH] OR "Bipolar and Related Disorders"[MeSH] OR "Bipolar Disorder"[MeSH] OR "Dissociative Disorders"[MeSH] OR "Mood Disorders"[MeSH] OR "Depressive Disorder"[MeSH] OR "Depression, Postpartum"[MeSH] OR "Depressive Disorder, Major"[MeSH] OR "Dysthymic Disorder"[MeSH] OR "Neurocognitive Disorders"[MeSH] OR "Neurotic Disorders"[MeSH] OR "Schizophrenia Spectrum and Other Psychotic Disorders"[MeSH] OR "Affective Disorders, Psychotic"[MeSH] OR "Psychotic Disorders"[MeSH] OR "Psychoses, Substance-Induced"[MeSH] OR "Schizophrenia"[MeSH] OR "Schizophrenia, Catatonic"[MeSH] OR "Schizophrenia, Disorganized"[MeSH] OR "Schizophrenia, Paranoid"[MeSH] OR "Shared Paranoid Disorder"[MeSH] OR "Suicide"[MeSH] OR "Suicide, Attempted"[MeSH] OR "Trauma and Stressor Related Disorders"[MeSH] OR "Psychological Trauma"[MeSH] OR "Stress Disorders, Post-Traumatic"[MeSH] OR "Stress Disorders, Traumatic, Acute"[MeSH] OR "Substance-Related Disorders"[MeSH] OR "Alcohol-Related Disorders"[MeSH] OR "Alcohol-Induced Disorders, Nervous System"[MeSH] OR "Psychoses, Alcoholic"[MeSH] OR "Psychoses, Substance-Induced"[MeSH] OR "Substance Abuse, Intravenous"[MeSH] OR ("mental"[tiab] AND ("disorder"[tiab] OR "disorders"[tiab] OR "illness"[tiab] OR "illnesses"[tiab])) OR "anxiety"[tiab] OR "bipolar"[tiab] OR "mood disorder"[tiab] OR "mood disorders"[tiab] OR "depression"[tiab] OR "depressive"[tiab] OR "depressed"[tiab] OR "neurotic"[tiab] OR "neurosis"[tiab] OR "PTSD"[tiab] OR "traumatic stress"[tiab] OR "trauma"[tiab] OR (("stress"[tiab] OR "distress"[tiab]) AND ("disorder"[tiab] OR "disorders"[tiab] OR "syndrome"[tiab] OR "syndromes"[tiab])) OR "schizophrenia"[tiab] OR "schizophrenic"[tiab] OR "psychotic"[tiab] OR "psychosis"[tiab] OR "psychoses"[tiab] OR "manic-depressive"[tiab] OR "manic depressive"[tiab] OR "paranoid"[tiab] OR "suicide"[tiab] OR "suicides"[tiab] OR "suicidal"[tiab] OR (("substance"[tiab] OR "drug"[tiab] OR "alcohol"[tiab]) AND ("disorder"[tiab] OR "disorders"[tiab] OR "use"[tiab] OR "using"[tiab] OR "abuse"[tiab])) OR "IDU"[tiab] OR "IDUs"[tiab] OR "Mentally Ill Persons"[MeSH] OR "Mentally Disabled Persons"[MeSH] OR (("mentally"[tiab] OR "mental"[tiab]) AND ("ill"[tiab] OR "disabled"[tiab] OR "handicapped"[tiab] OR "retarded"[tiab] OR "disability"[tiab] OR "disabilities"[tiab] OR "retardation"[tiab])) OR "Psychotherapy"[MeSH] OR "psychotherapy"[tiab] OR "psycho-therapy"[tiab] OR "psycho therapy"[tiab] OR (("cognitive"[tiab] OR "behavior"[tiab] OR "behaviour"[tiab] OR "behavioral"[tiab] OR "behavioural"[tiab]) AND "therapy"[tiab])) AND "humans"[MeSH Terms] |
| PsychINFO | (((mental or psychological or psychosocial or psycho-social or emotional) and (health or wellbeing or well-being or well being or resilience or resiliency or symptoms)) or ((mental or community) and (health service or health services or health center or health centers or health centre or health centres)) or ((mental health or psychiatric) and (recovery or nursing or rehabilitation)) or (mental and (disorder or disorders or illness or illnesses)) or (anxiety or bipolar or mood disorder or mood disorders or depression or depressive or depressed or neurotic or neurosis or PTSD or traumatic stress or trauma) or ((stress or distress) and (disorder or disorders or syndrome or syndromes)) or (schizophrenia or schizophrenic or psychotic or psychosis or psychoses or manic-depressive or manic depressive or paranoid or suicide or suicides or suicidal) or ((substance or drug or alcohol) and (disorder or disorders or abuse)) or (IDU or IDUs) or ((mentally or mental) and (ill or disabled or handicapped or retarded or disability or disabilities or retardation)) or (psychotherapy or psycho-therapy or psycho therapy) or ((cognitive or behavior or behaviour or behavioral or behavioural) and therapy)).ti.    **[OR]**    (((mental or psychological or psychosocial or psycho-social or emotional) and (health or wellbeing or well-being or well being or resilience or resiliency or symptoms)) or ((mental or community) and (health service or health services or health center or health centers or health centre or health centres)) or ((mental health or psychiatric) and (recovery or nursing or rehabilitation)) or (mental and (disorder or disorders or illness or illnesses)) or (anxiety or bipolar or mood disorder or mood disorders or depression or depressive or depressed or neurotic or neurosis or PTSD or traumatic stress or trauma) or ((stress or distress) and (disorder or disorders or syndrome or syndromes)) or (schizophrenia or schizophrenic or psychotic or psychosis or psychoses or manic-depressive or manic depressive or paranoid or suicide or suicides or suicidal) or ((substance or drug or alcohol) and (disorder or disorders or abuse )) or (IDU or IDUs) or ((mentally or mental) and (ill or disabled or handicapped or retarded or disability or disabilities or retardation)) or (psychotherapy or psycho-therapy or psycho therapy) or ((cognitive or behavior or behaviour or behavioral or behavioural) and therapy)).ab.    **[AND]**  (((community or lay or village or home or frontline or front-line or mental or nonphysician or untrained or trained or voluntary or nonmedical or non-medical or nonspecialist or non-specialist or nonprofessional or non-professional or paraprofessional or para-professional or extension or auxiliary) and (worker or workers or practitioners or representatives or advisors or counselors or health aides or guides or attendants or care givers or caregivers or consultants or volunteers or extenders)) or (promotora or promotores) or (patient and (navigators or navigator or navigation)) or (barefoot doctors or barefoot doctor) or ((task or tasks) and (shift or shifted or shifts or shifting or share or shared or sharing or delegation or delegating or substitution or substituting)) or ((shortage or substitution or substituted or substitute or substituting or substitutes or delegate or delegating or delegates or delegation or delegated) and (Physicians or Health Personnel or Nurses or Nurses, Community Health or Nurses, International or Nurses, Public Health or Personnel, Hospital or Hospital Volunteers or Medical Staff, Hospital or Nursing Staff, Hospital or physicians or doctors or trained personnel or health workforce or health care workforce or healthcare workforce or health workers or health care workers or healthcare workers or health care providers or health providers or healthcare providers or health professionals or healthcare professionals or health care professionals or health practitioners or nurses or psychologists or psychiatrists or clinicians)) or (nurse led or primary health care nurse or primary health care nurses or primary health care nursing) or (role and (nurse or nurses or nursing)) or (community based or (community and intervention) or self help group or self-help groups or support groups) or ((social or psychosocial) and (care or support))).af.  **[AND]**  (((emerging or developing or less developed or lesser developed or under developed or underdeveloped or under-developed or low income or low-income or lower income or lower-income or middle income or middle-income or underserved or under-served or under served or poor or poorer or poorest or deprived or transitional) and (country or countries or nation or nations or population or populations or economy or economies)) or world or (low gdp or low gnp or low gross domestic or low gross national or lower gdp or lower gnp or lower gross domestic or lower gross national or LMIC or LMICs or lami country or lami countries or third world) or (Africa or Asia or Caribbean or West Indies or South America or Latin America or Central America or Atlantic Islands or Commonwealth of Independent States or Pacific Islands or Indian Ocean Islands or Eastern Europe or Southern African Development Community or East African Community or West African Health Organisation or West African Health Organization or Pan American Health Organization or Pan American Health Organisation or PAHO) or (Afghanistan or Albania or Algeria or Angola or Armenia or Armenian or Azerbaijan or Bangladesh or Benin or Byelarus or Byelorussian or Belarus or Belorussian or Belorussia or Belize or Bhutan or Bolivia or Bosnia or Herzegovina or Hercegovina or Bosnia-Herzegovina or Botswana or Brasil or Brazil or Bulgaria or Burkina Faso or Burkina Fasso or Upper Volta or Burundi or Urundi or Cambodia or Khmer Republic or Kampuchea or Cameroon or Cameroons or Cameron or Cape Verde or Central African Republic or Chad or China or Colombia or Comoros or Comoro Islands or Comores or Mayotte or Congo or Zaire or Costa Rica or Cote d'Ivoire or Ivory Coast or Cuba or Czechoslovakia or Slovakia or Djibouti or French Somaliland or Dominica or Dominican Republic or East Timor or East Timur or Timor Leste or Ecuador or Egypt or El Salvador or Eritrea or Ethiopia or Fiji or Gabon or Gabonese Republic or Gambia or Gaza or Georgia Republic or Georgian Republic or Ghana or Gold Coast or Grenada or Guatemala or Guinea or Guiana or Guyana or Haiti or Honduras or India or Maldives or Indonesia or Iran or Iraq or Jamaica or Jordan or Kazakhstan or Kazakh or Kenya or Kiribati or Korea or Kosovo or Kyrgyzstan or Kirghizia or Kyrgyz Republic or Kirghiz or Kirgizstan or Lao PDR or Laos or Lebanon or Lesotho or Basutoland or Liberia or Libya or Macedonia or Madagascar or Malagasy Republic or Malaysia or Malaya or Malay or Sabah or Sarawak or Malawi or Nyasaland or Mali or Marshall Islands or Mauritania or Mauritius or Agalega Islands or Melanesia or Mexico or Micronesia or Middle East or Moldova or Moldovia or Moldovian or Mongolia or Montenegro or Morocco or Ifni or Mozambique or Myanmar or Myanma or Burma or Namibia or Nepal or Nicaragua or Niger or Nigeria or Muscat or Pakistan or Palau or Palestine or Panama or Paraguay or Peru or Philippines or Philipines or Phillipines or Phillippines or Romania or Rumania or Roumania or Rwanda or Ruanda or Saint Kitts or St Kitts or Nevis or Saint Lucia or St Lucia or Saint Vincent or St Vincent or Grenadines or Samoa or Samoan Islands or Navigator Island or Navigator Islands or Sao Tome or Senegal or Serbia or Montenegro or Sierra Leone or Sri Lanka or Ceylon or Solomon Islands or Somalia or Sudan or Suriname or Surinam or Swaziland or Syria or Syrian or Tajikistan or Tadzhikistan or Tadjikistan or Tadzhik or Tanzania or Thailand or Togo or Togolese Republic or Tonga or Tunisia or Turkey or Turkmenistan or Turkmen or Tuvalu or Uganda or Ukraine or Uzbekistan or Uzbek or Vanuatu or New Hebrides or Vietnam or Viet Nam or Viet-Nam or West Bank or Yemen or Yugoslavia or Zambia or Zimbabwe or Rhodesia)).af.  **Filters:**  Humans, English |
| CINAHL | (MH "Mental Health") OR (MH "Mental Health Services+") OR (MH "Community Mental Health Services+") OR (MH "Psychology+") OR ((TI mental [1] OR AB mental) [2] OR (TI psychiatric OR AB psychiatric) OR (TI psychological OR AB psychological) OR (TI psychosocial OR AB psychosocial) OR (TI emotional OR AB emotional)) AND ((TI health OR AB health) OR (TI wellbeing OR AB wellbeing) OR (TI well-being OR AB well-being) OR (TI recovery OR AB recovery) OR (TI resilience OR AB resilience) OR (TI rehabilitation OR AB rehabilitation)) (MH “Mental Disorders+”) OR (MH "Anxiety Disorders+") OR (TI Bipolar and Related Disorders OR AB Bipolar and Related Disorders) OR (MH "Bipolar Disorder+") OR (MH "Dissociative Disorders+") OR (MH "Affective Disorders+") OR (MH "Depression+") OR (MH "Depression, Postpartum") OR (TI Depressive Disorder, Major OR AB Depressive Disorder, Major) OR (MH "Dysthymic Disorder") OR (TI Neurocognitive Disorders OR AB Neurocognitive Disorders) OR (MH "Neurotic Disorders+") OR (TI Schizophrenia Spectrum and Other Psychotic Disorders OR AB Schizophrenia Spectrum and Other Psychotic Disorders) OR (MH "Affective Disorders, Psychotic+") OR (MH "Psychotic Disorders+") OR (MH "Psychoses, Substance-Induced+") OR (MH "Schizophrenia+") OR (TI Schizophrenia, Catatonic OR AB Schizophrenia, Catatonic) OR (TI Schizophrenia, Disorganized OR AB Schizophrenia, Disorganized) OR (TI Schizophrenia, Paranoid OR AB Schizophrenia, Paranoid) OR (TI Shared Paranoid Disorder OR AB Shared Paranoid Disorder) OR (MH "Suicide+") OR (MH "Suicide, Attempted") OR (TI Trauma and Stressor Related Disorders OR AB Trauma and Stressor Related Disorders) OR (MH "Psychological Trauma") OR (MH "Stress Disorders, Post-Traumatic+") OR (TI Stress Disorders, Traumatic, Acute OR AB Stress Disorders, Traumatic, Acute) OR (MH "Substance Use Disorders+") OR (MH "Alcohol-Related Disorders+") OR (MH "Alcohol-Induced Disorders, Nervous System+") OR (MH "Psychoses, Alcoholic+") OR (MH "Psychoses, Substance-Induced+") OR (MH "Substance Abuse, Intravenous") OR ((TI mental OR AB mental) AND ((TI disorder OR AB disorder) OR (TI disorders OR AB disorders) OR (TI illness OR AB illness) OR (TI illnesses OR AB illnesses))) OR (TI anxiety OR AB anxiety) OR (TI bipolar OR AB bipolar) OR (TI mood disorder OR AB mood disorder) OR (TI mood disorders OR AB mood disorders) OR (TI depression OR AB depression) OR (TI depressive OR AB depressive) OR (TI depressed OR AB depressed) OR (TI neurotic OR AB neurotic) OR (TI neurosis OR AB neurosis) OR (TI PTSD OR AB PTSD) OR (TI traumatic stress OR AB traumatic stress) OR (TI trauma OR AB trauma) OR (((TI stress OR AB stress) OR (TI distress OR AB distress) AND ((TI disorder OR AB disorder) OR (TI disorders OR AB disorders) OR (TI syndrome OR AB syndrome) OR (TI syndromes OR AB syndromes))) OR (TI schizophrenia OR AB schizophrenia) OR (TI schizophrenic OR AB schizophrenic) OR (TI psychotic OR AB psychotic) OR (TI psychosis OR AB psychosis) OR (TI psychoses OR AB psychoses) OR (TI manic-depressive OR AB manic-depressive) OR (TI paranoid OR AB paranoid) OR (TI suicide OR AB suicide) OR (TI suicides OR AB suicides) OR (TI suicidal OR AB suicidal) OR (((TI substance OR AB substance) OR (TI drug OR AB drug) OR (TI alcohol OR AB alcohol)) AND ((TI disorder OR AB disorder) OR (TI disorders OR AB disorders) OR (TI use OR AB use) OR (TI using OR AB using) OR (TI abuse OR AB abuse))) OR (TI IDU OR AB IDU) OR (TI IDUs OR AB IDUs) OR (TI Mentally Ill Persons OR AB Mentally Ill Persons) OR (MH "Mentally Disabled Persons") OR (((TI mentally OR AB mentally) OR (TI mental OR AB mental)) AND ((TI ill OR AB ill) OR (TI disabled OR AB disabled) OR (TI handicapped OR AB handicapped) OR (TI retarded OR AB retarded) OR (TI disability OR AB disability) OR (TI disabilities OR AB disabilities) OR (TI retardation OR AB retardation))) OR (MH "Psychotherapy+") OR (TI psychotherapy OR AB psychotherapy) OR (TI psycho-therapy OR AB psycho-therapy) OR (TI psycho therapy OR AB psycho therapy) OR (((TI cognitive OR AB cognitive) OR (TI behavior OR AB behavior) OR (TI behaviour OR AB behaviour) OR (TI behavioral OR AB behavioral) OR (TI behavioural OR AB behavioural)) AND (TI therapy OR AB therapy)) OR (MH "Human") AND (((TI community OR AB community) OR (TI lay OR AB lay) OR (TI village OR AB village) OR (TI frontline OR AB frontline) OR (TI front-line OR AB front-line) OR (TI mental OR AB mental) OR (TI nonphysician OR AB nonphysician) OR (TI untrained OR AB untrained) OR (TI trained OR AB trained) OR (TI voluntary OR AB voluntary) OR (TI nonphysician OR AB nonphysician) OR (TI nonmedical OR AB nonmedical) OR (TI non-medical OR AB non-medical) OR (TI nonspecialist OR AB nonspecialist) OR (TI non-specialist OR AB non-specialist) OR (TI nonprofessional OR AB nonprofessional) OR (TI paraprofessional OR AB paraprofessional) OR (TI para-professional OR AB para-profesional) OR (TI extension OR AB extension) OR (TI auxiliary OR AB auxiliary)) AND ((TI worker OR AB worker) OR (TI workers OR AB workers) OR (TI practitioners OR AB practitioners) OR (TI representatives OR AB representatives) OR (TI advisors OR AB advisors) OR (MH "Counselors+") OR (TI health aides OR AB health aides) OR (TI guides OR AB guides) OR (TI attendants OR AB attendants) OR (MH "Caregivers") OR (MH "Consultants+") OR (TI volunteers OR AB volunteers) OR (TI extenders OR AB extenders))) OR (MH "Volunteer Workers") OR (TI promotora OR AB promotora) OR (TI promotores OR AB promotores) OR ((TI patient OR AB patient) AND ((TI navigators OR AB navigators) OR (TI navigator OR AB navigator) OR (TI navigation OR AB navigation))) OR (TI barefoot doctors OR AB barefoot doctors) OR (TI barefoot doctor OR AB barefoot doctor) OR (((TI task OR AB task) OR (TI tasks OR AB tasks)) AND ((TI shift OR AB shift) OR (TI shifted OR AB shifted) OR (TI shifts OR AB shifts) OR (TI shifting OR AB shifting) OR (TI share OR AB share) OR (TI shared OR AB shared) OR (TI sharing OR AB sharing) OR (TI delegation OR AB delegation) OR (TI delegating OR AB delegating) OR (TI substitution OR AB substitution) OR (TI substituting OR AB substituting))) OR (((TI shortage OR AB shortage) OR (TI substitution OR AB substitution) OR (TI substituted OR AB substituted) OR (TI substitute OR AB substitute) OR (TI substituting OR AB substituting) OR (TI substitutes OR AB substitutes) OR (TI delegate OR AB delegate) OR (TI delegating OR AB delegating) OR (TI delegates OR AB delegates) OR (TI delegation OR AB delegation) OR (TI delegated OR AB delegated)) AND ((MH "Health Personnel+") OR (MH "Nurses+") OR (TI community health OR AB community health) OR (MH "Personnel, Health Facility+") OR (TI hospital volunteers OR AB hospital volunteers) OR (MH "Medical Staff, Hospital+") OR (MH "Nursing Staff, Hospital") OR (MH “Physicians+”) OR (TI trained personnel OR AB trained personnel) OR (TI health workforce OR AB health workforce) OR (TI health care workforce OR AB health care workforce) OR (TI healthcare workforce OR AB healthcare workforce) OR (MH "Health Personnel+") OR (TI health providers OR AB health providers) OR (TI healthcare providers OR AB healthcare providers) OR (TI health professionals OR AB health professionals) OR (TI healthcare professionals OR AB healthcare professionals) OR (TI health care professionals OR AB health care professionals) OR (TI health practitioners OR AB health practitioners) OR (MH “Nurses+”) OR (MH “Psychologists”) OR (MH "Psychiatrists") OR (TI clinicians OR AB clinicians))) OR (TI nurse led OR AB nurse led) OR (TI primary health care nurse OR AB primary health care nurse) OR (TI primary health care nurses OR AB primary health care nurses) OR (TI primary health care nursing OR AB primary health care nursing) OR ((TI role OR AB role) AND (MH “Nurses+”)) OR (TI community based OR AB community based) OR ((TI community OR AB community) AND (TI intervention OR AB intervention)) OR (MH "Support Groups+") OR (MH "Support, Psychosocial+") AND (MH "Low and Middle Income Countries") OR (((TI emerging OR AB emerging) OR (TI developing OR AB developing) OR (TI less developed OR AB less developed) OR (TI lesser developed OR AB lesser developed) OR (TI under developed OR AB under developed) OR (TI underdeveloped OR AB underdeveloped) OR (TI under-developed OR AB under-developed) OR (TI low income OR AB low income) OR (TI low-income OR AB low-income) OR (TI lower income OR AB lower income) OR (TI lower-income OR AB lower-income) OR (TI middle income OR AB middle income) OR (TI middle-income OR AB middle-income) OR (TI underserved OR AB underserved) OR (TI under-served OR AB under-served) OR (TI under served OR AB under served) OR (TI poor OR AB poor) OR (TI poorer OR AB poorer) OR (TI poorest OR AB poorest) OR (TI deprived OR AB deprived) OR (TI transitional OR AB transitional)) AND ((TI country OR AB country) OR (TI countries OR AB countries) OR (TI nation OR AB nation) OR (TI nations OR AB nations) OR (TI population OR AB population) OR (TI populations OR AB populations) OR (TI economy OR AB economy) OR (TI economies OR AB economies))) OR (TI world OR AB world) OR (TI low gdp OR AB low gdp) OR (TI low gnp OR AB low gnp) OR (TI low gross domestic OR AB low gross domestic) OR (TI low gross national OR AB low gross national) OR (TI lower gdp OR AB lower gdp) OR (TI lower gnp OR AB lower gnp) OR (TI lower gross domestic OR AB lower gross domestic) OR (TI lower gross national OR AB lower gross national) OR (TI lmic OR AB lmic) OR (TI lmics OR AB lmics) OR (MH "Developing Countries") OR (MH “Africa+”) OR (MH “Asia+”) OR (MH “West Indies+”) OR (MH “South America+”) OR (MH “Latin America”) OR (MH “Central America+”) OR (MH “Atlantic Islands+”) OR (MH "Commonwealth of Independent States+") OR (MH “Pacific Islands+”) OR (MH “Indian Ocean Islands+”) OR (MH “Europe, Eastern+”) OR (TI southern African development community OR AB southern African development community) OR (TI east African community OR AB east African community) OR (TI west African health organization OR AB west African health organization) OR (TI west African health organisation OR AB west African health organisation) OR (MH "Pan American Health Organization") OR (MH “Afghanistan”) OR (MH “Albania”) OR (MH “Algeria”) OR (MH “Angola”) OR (MH “Armenia”) OR (MH “Azerbaijan”) OR (MH “Bangladesh”) OR (MH “Benin”) OR (MH “Byelarus”) OR (MH “Belize”) OR (MH “Bhutan”) OR (MH “Bolivia”) OR (MH “Bosnia-Herzegovina”) OR (MH “Botswana”) OR (MH “Brazil”) OR (MH “Bulgaria”) OR (MH “Burkina Faso”) OR (TI upper volta OR AB upper volta) OR (MH “Burundi”) OR (MH “Cambodia”) OR (MH “Cameroon”) OR (MH “Cape Verde”) OR (MH “Central African Republic”) OR (MH “Chad”) OR (MH “China+”) OR (MH “Colombia”) OR (TI comoros OR AB comoros) OR (TI Comoro islands OR AB Comoro islands) OR (TI mayotte OR AB mayotte) OR (MH “Congo”) OR (MH “Democratic Republic of the Congo”) OR (MH “Costa Rica”) OR (MH “Cote d’Ivoire”) OR (MH “Cuba”) OR (MH “Czechoslovakia+”) OR (MH “Slovakia”) OR (MH “Djibouti”) OR (MH “Dominica”) OR (MH “Dominican Republic”) OR (MH “East Timor”) OR (MH “Timor”) OR (MH “Ecuador”) OR (MH “Egypt”) OR (MH “El Salvador”) OR (MH “Eritrea”) OR (MH “Ethiopia”) OR (MH “Melanesia”) OR (MH “Gabon”) OR (MH “Gambia”) OR (TI gaza OR AB gaza) OR (MH "Georgia (Republic)") OR (MH “Ghana”) OR (TI gold coast OR AB gold coast) OR (TI grenada OR AB grenada) OR (MH “Guatemala”) OR (MH “Guinea”) OR (MH “French Guiana”) OR (MH “Guyana”) OR (MH “Haiti”) OR (MH “Honduras”) OR (MH “India”) OR (TI Maldives OR AB Maldives) OR (MH “Indonesia”) OR (MH “Iran”) OR (MH “Iraq”) OR (MH “Jamaica”) OR (MH “Jordan”) OR (MH “Kazakhstan”) OR (MH “Kenya”) OR (TI Kiribati OR AB Kiribati) OR (MH “Korea”) OR (MH “Yugoslavia”) OR (MH “Krygyzstan”) OR (TI Kirghizia OR AB Kirghizia) OR (TI Kyrgyz republic OR AB Kyrgyz republic) OR (TI kirghiz OR AB kirghiz) OR (MH “Laos”) OR (MH “Lebanon”) OR (MH “Lesotho”) OR (TI Basutoland OR AB Basutoland) OR (MH “Liberia”) OR (MH “Libya”) OR (MH “Macedonia (Republic)”) OR (MH “Madagascar”) OR (MH “Malaysia”) OR (TI malay OR AB malay) OR (TI sabah OR AB sabah) OR (TI Sarawak OR AB Sarawak) OR (MH “Malawi”) OR (MH “Mali”) OR (TI Marshall Islands OR AB Marshall Islands) OR (MH “Mauritania”) OR (TI agalega islands OR AB agalega islands) OR (MH “Mexico”) OR (MH “Micronesia+”) OR (MH “Middle East+”) OR (MH “Moldova”) OR (MH “Mongolia”) OR (MH “Morocco”) OR (TI ifni OR AB ifni) OR (MH “Mozambique”) OR (MH “Myanmar”) OR (MH “Namibia”) OR (MH “Nepal”) OR (MH “Nicaragua”) OR (MH “Niger”) OR (MH “Nigeria”) OR (MH “Oman”) OR (MH “Pakistan”) OR (TI palau OR AB palau) OR (TI Palestine OR AB Palestine) OR (MH “Panama+”) OR (MH “Paraguay”) OR (MH “Peru”) OR (MH “Philippines”) OR (MH “Romania”) OR (MH “Rwanda”) OR (TI saint kitts OR AB saint kitts) OR (TI st kitts OR AB st kitts) OR (TI nevis OR AB nevis) OR (TI saint lucia OR AB saint lucia) OR (TI st lucia OR AB st lucia) OR (TI saint Vincent OR AB saint Vincent) OR (TI st Vincent OR AB st Vincent) OR (TI grenadines OR AB grenadines) OR (MH “Samoa+”) OR (TI samoan islands OR AB samoan islands) OR (TI navigator island OR AB navigator island) OR (TI navigator islands OR AB navigator islands) OR (TI sao tome OR AB sao tome) OR (MH “Senegal”) OR (MH “Serbia”) OR (TI Montenegro OR AB Montenegro) OR (MH “Sierra Leone”) OR (MH “Sri Lanka”) OR (TI Solomon islands OR AB Solomon islands) OR (MH “Somalia”) OR (MH “Sudan”) OR (MH “Suriname”) OR (MH “Swaziland”) OR (MH “Syria”) OR (MH “Tajikistan”) OR (MH “Tanzania”) OR (MH “Thailand”) OR (MH “Togo”) OR (MH “Polynesia”) OR (MH “Tonga”) OR (MH “Tunisia”) OR (MH “Turkmenistan”) OR (TI Tuvalu OR AB Tuvalu) OR (MH “Uganda”) OR (MH “Ukraine”) OR (MH “Uzbekistan”) OR (TI Vanuatu OR AB Vanuatu) OR (MH “Vietnam”) OR (TI west bank OR AB west bank) OR (MH “Yemen”) OR (MH “Zambia”) OR (MH “Zimbabwe”) |
| Embase | ('mental':ab,ti OR 'psychological':ab,ti OR 'psychosocial':ab,ti OR 'psycho-social':ab,ti OR 'emotional':ab,ti) AND ('health':ab,ti OR 'wellbeing':ab,ti OR 'well-being':ab,ti OR 'well being':ab,ti OR 'resilience':ab,ti OR 'resiliency':ab,ti OR 'symptoms':ab,ti) OR (('mental':ab,ti OR 'community':ab,ti) AND ('health service':ab,ti OR 'health services':ab,ti OR 'health center':ab,ti OR 'health centers':ab,ti OR 'health centre':ab,ti OR 'health centres':ab,ti)) OR (('mental health':ab,ti OR 'psychiatric':ab,ti) AND ('recovery':ab,ti OR 'nursing':ab,ti OR 'rehabilitation':ab,ti)) OR ('mental':ab,ti AND ('disorder':ab,ti OR 'disorders':ab,ti OR 'illness':ab,ti OR 'illnesses':ab,ti)) OR 'anxiety':ab,ti OR 'bipolar':ab,ti OR 'mood disorder':ab,ti OR 'mood disorders':ab,ti OR 'depression':ab,ti OR 'depressive':ab,ti OR 'depressed':ab,ti OR 'neurotic':ab,ti OR 'neurosis':ab,ti OR 'ptsd':ab,ti OR 'traumatic stress':ab,ti OR 'trauma':ab,ti OR (('stress':ab,ti OR 'distress':ab,ti) AND ('disorder':ab,ti OR 'disorders':ab,ti OR 'syndrome':ab,ti OR 'syndromes':ab,ti)) OR 'schizophrenia':ab,ti OR 'schizophrenic':ab,ti OR 'psychotic':ab,ti OR 'psychosis':ab,ti OR 'psychoses':ab,ti OR 'manic-depressive':ab,ti OR 'manic depressive':ab,ti OR 'paranoid':ab,ti OR 'suicide':ab,ti OR 'suicides':ab,ti OR 'suicidal':ab,ti OR (('substance':ab,ti OR drug:ab,ti OR alcohol:ab,ti) AND ('disorder':ab,ti OR 'disorders':ab,ti OR 'abuse':ab,ti)) OR 'idu':ab,ti OR 'idus':ab,ti OR (('mentally':ab,ti OR 'mental':ab,ti) AND ('ill':ab,ti OR 'disabled':ab,ti OR 'handicapped':ab,ti OR 'retarded':ab,ti OR 'disability':ab,ti OR 'disabilities':ab,ti OR 'retardation':ab,ti)) OR 'psychotherapy':ab,ti OR 'psycho-therapy':ab,ti OR 'psycho therapy':ab,ti OR (('cognitive':ab,ti OR 'behavior':ab,ti OR 'behaviour':ab,ti OR 'behavioral':ab,ti OR 'behavioural':ab,ti) AND 'therapy':ab,ti)  **[AND]**    ('community health workers':ab,ti OR 'allied health personnel':ab,ti OR 'home health aides':ab,ti OR 'nursing assistants':ab,ti OR 'psychiatric aides':ab,ti OR 'hospital volunteers':ab,ti OR 'community':ab,ti OR 'lay':ab,ti OR 'village':ab,ti OR 'home':ab,ti OR 'frontline':ab,ti OR 'front-line':ab,ti OR 'mental':ab,ti OR 'nonphysician':ab,ti OR 'untrained':ab,ti OR 'trained':ab,ti OR 'voluntary':ab,ti OR 'nonmedical':ab,ti OR 'non-medical':ab,ti OR 'nonspecialist':ab,ti OR 'non-specialist':ab,ti OR 'nonprofessional':ab,ti OR 'non-professional':ab,ti OR 'paraprofessional':ab,ti OR 'para-professional':ab,ti OR 'extension':ab,ti OR 'auxiliary':ab,ti OR 'worker':ab,ti OR 'workers':ab,ti OR 'practitioners':ab,ti OR 'representatives':ab,ti OR 'advisors':ab,ti OR 'counselors':ab,ti OR 'health aides':ab,ti OR 'guides':ab,ti OR 'attendants':ab,ti OR 'care givers':ab,ti OR 'caregivers':ab,ti OR 'consultants':ab,ti OR 'volunteers':ab,ti OR 'extenders':ab,ti OR 'promotora':ab,ti OR 'promotores':ab,ti OR (patient:ab,ti AND navigat*:ab,ti) OR 'barefoot doctors':ab,ti OR 'barefoot doctor':ab,ti) AND ('task':ab,ti OR 'tasks':ab,ti) AND ('shift':ab,ti OR 'shifted':ab,ti OR 'shifts':ab,ti OR 'shifting':ab,ti OR 'share':ab,ti OR 'shared':ab,ti OR 'sharing':ab,ti OR 'shortage':ab,ti OR 'substitution':ab,ti OR 'substituted':ab,ti OR 'substitute':ab,ti OR 'substituting':ab,ti OR 'substitutes':ab,ti OR 'delegate':ab,ti OR 'delegating':ab,ti OR 'delegates':ab,ti OR 'delegation':ab,ti OR 'delegated':ab,ti) AND ('health personnel':ab,ti OR 'nurses, community health':ab,ti OR 'nurses, international':ab,ti OR 'nurses, public health':ab,ti OR 'personnel, hospital':ab,ti OR 'hospital volunteers':ab,ti OR 'medical staff, hospital':ab,ti OR 'nursing staff, hospital':ab,ti OR 'physicians':ab,ti OR 'doctors':ab,ti OR 'trained personnel':ab,ti OR 'health workforce':ab,ti OR 'health care workforce':ab,ti OR 'healthcare workforce':ab,ti OR 'health workers':ab,ti OR 'health care workers':ab,ti OR 'healthcare workers':ab,ti OR 'health care providers':ab,ti OR 'health providers':ab,ti OR 'healthcare providers':ab,ti OR 'health professionals':ab,ti OR 'healthcare professionals':ab,ti OR 'health care professionals':ab,ti OR 'health practitioners':ab,ti OR 'nurses':ab,ti OR 'psychologists':ab,ti OR 'psychiatrists':ab,ti OR 'clinicians':ab,ti OR 'nurse led':ab,ti OR 'primary health care nurse':ab,ti OR 'primary health care nurses':ab,ti OR 'primary health care nursing':ab,ti OR 'nursing care':ab,ti OR 'nursing':ab,ti) OR 'self help group':ab,ti OR 'self-help groups':ab,ti OR 'support groups':ab,ti  **[AND]**  ('emerging':ab,ti OR 'developing':ab,ti OR 'less developed':ab,ti OR 'lesser developed':ab,ti OR 'under developed':ab,ti OR 'underdeveloped':ab,ti OR 'under-developed':ab,ti OR 'low income':ab,ti OR 'low-income':ab,ti OR 'lower income':ab,ti OR 'lower-income':ab,ti OR 'middle income':ab,ti OR 'middle-income':ab,ti OR 'underserved':ab,ti OR 'under-served':ab,ti OR 'under served':ab,ti OR 'poor':ab,ti OR 'poorer':ab,ti OR 'poorest':ab,ti OR 'deprived':ab,ti OR 'transitional':ab,ti) AND ('country':ab,ti OR 'countries':ab,ti OR 'nation':ab,ti OR 'nations':ab,ti OR 'population':ab,ti OR 'populations':ab,ti OR 'economy':ab,ti OR 'economies':ab,ti) OR 'world':ab,ti OR 'low gdp':ab,ti OR 'low gnp':ab,ti OR 'low gross domestic':ab,ti OR 'low gross national':ab,ti OR 'lower gdp':ab,ti OR 'lower gnp':ab,ti OR 'lower gross domestic':ab,ti OR 'lower gross national':ab,ti OR 'lmic':ab,ti OR 'lmics':ab,ti OR 'lami country':ab,ti OR 'lami countries':ab,ti OR 'third world':ab,ti OR 'africa':ab,ti OR 'asia':ab,ti OR 'caribbean':ab,ti OR 'west indies':ab,ti OR 'south america':ab,ti OR 'latin america':ab,ti OR 'central america':ab,ti OR 'atlantic islands':ab,ti OR 'commonwealth of independent states':ab,ti OR 'pacific islands':ab,ti OR 'indian ocean islands':ab,ti OR 'eastern europe':ab,ti OR 'southern african development community':ab,ti OR 'east african community':ab,ti OR 'west african health organisation':ab,ti OR 'west african health organization':ab,ti OR 'pan american health organization':ab,ti OR 'pan american health organisation':ab,ti OR 'paho':ab,ti OR 'afghanistan':ab,ti OR 'albania':ab,ti OR 'algeria':ab,ti OR 'angola':ab,ti OR 'armenia':ab,ti OR 'armenian':ab,ti OR 'azerbaijan':ab,ti OR 'bangladesh':ab,ti OR 'benin':ab,ti OR 'byelarus':ab,ti OR 'byelorussian':ab,ti OR 'belarus':ab,ti OR 'belorussian':ab,ti OR 'belorussia':ab,ti OR 'belize':ab,ti OR 'bhutan':ab,ti OR 'bolivia':ab,ti OR 'bosnia':ab,ti OR 'herzegovina':ab,ti OR 'hercegovina':ab,ti OR 'bosnia-herzegovina':ab,ti OR 'botswana':ab,ti OR 'brasil':ab,ti OR 'brazil':ab,ti OR 'bulgaria':ab,ti OR 'burkina faso':ab,ti OR 'burkina fasso':ab,ti OR 'upper volta':ab,ti OR 'burundi':ab,ti OR 'urundi':ab,ti OR 'cambodia':ab,ti OR 'khmer republic':ab,ti OR 'kampuchea':ab,ti OR 'cameroon':ab,ti OR 'cameroons':ab,ti OR 'cameron':ab,ti OR 'cape verde':ab,ti OR 'central african republic':ab,ti OR 'chad':ab,ti OR 'china':ab,ti OR 'colombia':ab,ti OR 'comoros':ab,ti OR 'comoro islands':ab,ti OR 'comores':ab,ti OR 'mayotte':ab,ti OR 'congo':ab,ti OR 'zaire':ab,ti OR 'costa rica':ab,ti OR 'cote divoire':ab,ti OR 'ivory coast':ab,ti OR 'cuba':ab,ti OR 'czechoslovakia':ab,ti OR 'slovakia':ab,ti OR 'djibouti':ab,ti OR 'french somaliland':ab,ti OR 'dominica':ab,ti OR 'dominican republic':ab,ti OR 'east timor':ab,ti OR 'east timur':ab,ti OR 'timor leste':ab,ti OR 'ecuador':ab,ti OR 'egypt':ab,ti OR 'el salvador':ab,ti OR 'eritrea':ab,ti OR 'ethiopia':ab,ti OR 'fiji':ab,ti OR 'gabon':ab,ti OR 'gabonese republic':ab,ti OR 'gambia':ab,ti OR 'gaza':ab,ti OR 'georgia republic':ab,ti OR 'georgian republic':ab,ti OR 'ghana':ab,ti OR 'gold coast':ab,ti OR 'grenada':ab,ti OR 'guatemala':ab,ti OR 'guinea':ab,ti OR 'guiana':ab,ti OR 'guyana':ab,ti OR 'haiti':ab,ti OR 'honduras':ab,ti OR 'india':ab,ti OR 'maldives':ab,ti OR 'indonesia':ab,ti OR 'iran':ab,ti OR 'iraq':ab,ti OR 'jamaica':ab,ti OR 'jordan':ab,ti OR 'kazakhstan':ab,ti OR 'kazakh':ab,ti OR 'kenya':ab,ti OR 'kiribati':ab,ti OR 'korea':ab,ti OR 'kosovo':ab,ti OR 'kyrgyzstan':ab,ti OR 'kirghizia':ab,ti OR 'kyrgyz republic':ab,ti OR 'kirghiz':ab,ti OR 'kirgizstan':ab,ti OR 'lao pdr':ab,ti OR 'laos':ab,ti OR 'lebanon':ab,ti OR 'lesotho':ab,ti OR 'basutoland':ab,ti OR 'liberia':ab,ti OR 'libya':ab,ti OR 'macedonia':ab,ti OR 'madagascar':ab,ti OR 'malagasy republic':ab,ti OR 'malaysia':ab,ti OR 'malaya':ab,ti OR 'malay':ab,ti OR 'sabah':ab,ti OR 'sarawak':ab,ti OR 'malawi':ab,ti OR 'nyasaland':ab,ti OR 'mali':ab,ti OR 'marshall islands':ab,ti OR 'mauritania':ab,ti OR 'mauritius':ab,ti OR 'agalega islands':ab,ti OR 'melanesia':ab,ti OR 'mexico':ab,ti OR 'micronesia':ab,ti OR 'middle east':ab,ti OR 'moldova':ab,ti OR 'moldovia':ab,ti OR 'moldovian':ab,ti OR 'mongolia':ab,ti OR 'morocco':ab,ti OR 'ifni':ab,ti OR 'mozambique':ab,ti OR 'myanmar':ab,ti OR 'myanma':ab,ti OR 'burma':ab,ti OR 'namibia':ab,ti OR 'nepal':ab,ti OR 'nicaragua':ab,ti OR 'niger':ab,ti OR 'nigeria':ab,ti OR 'muscat':ab,ti OR 'pakistan':ab,ti OR 'palau':ab,ti OR 'palestine':ab,ti OR 'panama':ab,ti OR 'paraguay':ab,ti OR 'peru':ab,ti OR 'philippines':ab,ti OR 'philipines':ab,ti OR 'phillipines':ab,ti OR 'phillippines':ab,ti OR 'romania':ab,ti OR 'rumania':ab,ti OR 'roumania':ab,ti OR 'rwanda':ab,ti OR 'ruanda':ab,ti OR 'saint kitts':ab,ti OR 'st kitts':ab,ti OR 'nevis':ab,ti OR 'saint lucia':ab,ti OR 'st lucia':ab,ti OR 'saint vincent':ab,ti OR 'st vincent':ab,ti OR 'grenadines':ab,ti OR 'samoa':ab,ti OR 'samoan islands':ab,ti OR 'navigator island':ab,ti OR 'navigator islands':ab,ti OR 'sao tome':ab,ti OR 'senegal':ab,ti OR 'serbia':ab,ti OR 'montenegro':ab,ti OR 'sierra leone':ab,ti OR 'sri lanka':ab,ti OR 'ceylon':ab,ti OR 'solomon islands':ab,ti OR 'somalia':ab,ti OR 'sudan':ab,ti OR 'suriname':ab,ti OR 'surinam':ab,ti OR 'swaziland':ab,ti OR 'syria':ab,ti OR 'syrian':ab,ti OR 'tajikistan':ab,ti OR 'tadzhikistan':ab,ti OR 'tadjikistan':ab,ti OR 'tadzhik':ab,ti OR 'tanzania':ab,ti OR 'thailand':ab,ti OR 'togo':ab,ti OR 'togolese republic':ab,ti OR 'tonga':ab,ti OR 'tunisia':ab,ti OR 'turkey':ab,ti OR 'turkmenistan':ab,ti OR 'turkmen':ab,ti OR 'tuvalu':ab,ti OR 'uganda':ab,ti OR 'ukraine':ab,ti OR 'uzbekistan':ab,ti OR 'uzbek':ab,ti OR 'vanuatu':ab,ti OR 'new hebrides':ab,ti OR 'vietnam':ab,ti OR 'viet nam':ab,ti OR 'viet-nam':ab,ti OR 'west bank':ab,ti OR 'yemen':ab,ti OR 'yugoslavia':ab,ti OR 'zambia':ab,ti OR 'zimbabwe':ab,ti OR 'rhodesia':ab,ti  **Filters:**  Humans, English Only |
