# Supplementary material for: Barriers and facilitators to implementation of evidence-based task-sharing mental health interventions in low- and middle-income countries: a systematic review using implementation science frameworks
Source: Implement Sci. 2022 Jan 12;17:4. doi: 10.1186/s13012-021-01179-z (PMC8756725; doi:10.1186/s13012-021-01179-z)
Supplement: Supplementary file 1 — Additional file 1: A. Barriers and facilitators to implementation of evidence-based task-sharing mental health interventions in low- and middle-income countries: A systematic review using implementation science frameworks: PRISMA Checklist. B. Barriers and facilitators to implementation of evidence-based task-sharing mental health interventions in low- and middle-income countries: A systematic review using implementation science frameworks: Search Syntax. C. Barriers and facilitators to implementation of evidence-based task-sharing mental health interventions in low- and middle-income countries: A systematic review using implementation science frameworks: Quality Assessment Information by Study Type. [file 13012_2021_1179_MOESM1_ESM.zip › EBP_TSMH SysRev AddFile 1C Quality AssessmentR4.docx]

**Additional File 1C for *Barriers and facilitators to implementation of evidence-based task-sharing mental health interventions in low- and middle-income countries: A systematic review using implementation science frameworks*: Quality Assessment Information by Study Type**

*Qualitative studies*

| Author(s) and Year | Critical Appraisal Tool Type and Corresponding Criteria for Study | | | | | | | | | | | Summary of Critical Appraisal for Study | | | | | Criteria from the Text & Opinion Critical Appraisal Tool Used | | | Summary of Critical Appraisal for Discussion | | | Overall Appraisal |
| --- | --- | --- | --- | --- | --- | --- | --- | --- | --- | --- | --- | --- | --- | --- | --- | --- | --- | --- | --- | --- | --- | --- | --- |
|  | Critical Appraisal Tool Used (Additional Details) | Congruity between the stated philosophical perspective and the research methodology | Congruity between the research methodology and the research question or objectives | Congruity between the research methodology and the methods used to collect data | Congruity between the research methodology and the representation and analysis of data | Congruity between the research methodology and the interpretation of results | Statement locating the researcher culturally or theoretically | Addressed influence of the researcher on the research, and vice- versa | Adequate representation of participants and their voices | Evidence of ethical approval by an appropriate body | Conclusions drawn in the research report flow from the analysis, or interpretation, of the data | Total Number of Criteria | Number of Criteria Met | Number of Criteria Not Met | Number of Criteria Unclear | Number of Criteria Not Applicable | Source of the opinion clearly identified | The stated position is the result of an analytical process and there is logic in the opinion expressed | Any incongruence with the literature/sources is logically defended | Number of Criteria Met | Number of Criteria Unclear | Number of Criteria Not Met |  |
| Pacichana-Quinayáz et al. (2016) | Qualitative | Y | Y | Y | Y | Y | Y | U | Y | Y | Y | 10 | 9 | 0 | 1 | 0 | 1 | 1 | 1 | 3 | 0 | 0 | Include |
| Chatterjee et al. (2008) | Qualitative (with process indicators) | Y | Y | Y | Y | Y | U | U | Y | Y | Y | 10 | 8 | 0 | 2 | 0 | 1 | 1 | 1 | 3 | 0 | 0 | Include |
| Shinde et al. (2013) | Qualitative | Y | Y | Y | Y | Y | U | U | Y | Y | Y | 10 | 8 | 0 | 2 | 0 | 1 | 1 | 1 | 3 | 0 | 0 | Include |
| Spagnolo et al. (2018) | Qualitative | Y | Y | Y | Y | Y | Y | U | Y | Y | Y | 10 | 9 | 0 | 1 | 0 | 1 | 1 | 1 | 3 | 0 | 0 | Include |
| Tewari et al. (2017) | Qualitative (with process indicators) | U | Y | Y | Y | Y | Y | N | Y | Y | Y | 10 | 8 | 1 | 1 | 0 | 1 | 1 | 1 | 3 | 0 | 0 | Include |
| Shields et al. (2016) | Qualitative | Y | Y | Y | Y | Y | U | U | Y | Y | Y | 10 | 8 | 0 | 2 | 0 | 1 | 1 | 1 | 3 | 0 | 0 | Include |
| Abas et al. (2016) | Qualitative (with process indicators) | U | Y | Y | Y | Y | Y | N | Y | Y | Y | 10 | 8 | 1 | 1 | 0 | 1 | 1 | 1 | 3 | 0 | 0 | Include |
| Chibanda et al. (2017) | Qualitative | U | Y | Y | Y | Y | N | Y | Y | Y | Y | 10 | 8 | 1 | 1 | 0 | 1 | 1 | 1 | 3 | 0 | 0 | Include |
| Woods-Jaeger et al. (2017) | Qualitative | Y | Y | Y | Y | Y | Y | Y | Y | Y | Y | 10 | 0 | 0 | 0 | 0 | 1 | 1 | 1 | 3 | 0 | 0 | Include |
| Udedi et al. (2018) | Qualitative | Y | Y | Y | Y | Y | N | Y | Y | Y | Y | 10 | 9 | 1 | 0 | 0 | 1 | 1 | 1 | 3 | 0 | 0 | Include |
| Adewuya et al. (2017) | Qualitative | Y | Y | Y | Y | Y | N | N | Y | Y | Y | 10 | 8 | 2 | 0 | 0 | 1 | 1 | 1 | 3 | 0 | 0 | Include |
| Petersen et al. (2012a) | Qualitative | Y | Y | Y | Y | Y | Y | NA | Y | Y | Y | 10 | 9 | 0 | 0 | 1 | 1 | 1 | 1 | 3 | 0 | 0 | Include |
| Everitt-Penhale et al. (2019) | Qualitative | Y | Y | Y | Y | Y | U | Y | Y | Y | Y | 10 | 9 | 0 | 1 | 0 | 1 | 1 | 1 | 3 | 0 | 0 | Include |
| Munodawafa et al. (2017) | Qualitative | Y | Y | Y | Y | Y | U | Y | Y | Y | Y | 10 | 9 | 0 | 1 | 0 | 1 | 1 | 1 | 3 | 0 | 0 | Include |
| Nyatsanza et al. (2016)* | Qualitative | Y | Y | Y | Y | Y | U | U | Y | Y | Y | 10 | 8 | 0 | 2 | 0 | 1 | 1 | 1 | 3 | 0 | 0 | Include |
| Zafar et al. (2014) | Qualitative | Y | Y | Y | Y | Y | Y | NA | Y | Y | Y | 10 | 9 | 0 | 0 | 1 | 1 | 1 | 1 | 3 | 0 | 0 | Include |
| Mendenhall et al. (2014) | Qualitative | Y | Y | Y | Y | Y | Y | NA | Y | Y | Y | 10 | 9 | 0 | 0 | 1 | 1 | 1 | 1 | 3 | 0 | 0 | Include |
| Gureje et al. (2015) | Qualitative | NA | Y | Y | Y | Y | NA | NA | Y | Y | Y | 10 | 7 | 0 | 0 | 3 | 1 | 1 | 1 | 3 | 0 | 0 | Include |
| Hanlon et al. (2014) | Qualitative (Situational Analysis tool & MH system data) | NA | Y | Y | Y | Y | Y | NA | Y | Y | Y | 10 | 8 | 0 | 0 | 2 | 1 | 1 | 1 | 3 | 0 | 0 | Include |

Note: Y = yes, N = no, U = unclear, NA = not applicable.

*Randomized controlled trials*

| Author(s) and Year | Critical Appraisal Tool Type and Corresponding Criteria for Study | | | | | | | | | | | | | | Summary of Critical Appraisal for Study | | | | | Criteria from the Text & Opinion Critical Appraisal Tool Used | | | Summary of Critical Appraisal for Discussion | | | Overall Appraisal |
| --- | --- | --- | --- | --- | --- | --- | --- | --- | --- | --- | --- | --- | --- | --- | --- | --- | --- | --- | --- | --- | --- | --- | --- | --- | --- | --- |
|  | Critical Appraisal Tool Used (Additional Details) | True randomization was used for assignment of participants to treatment groups | Allocation to treatment groups was concealed | Treatment groups were similar at the baseline | Participants were blind to treatment assignment | Those delivering treatment were blind to treatment assignment | Outcomes assessors were blind to treatment assignment | Treatment groups were treated identically other than the intervention of interest | Follow up was complete and if not, differences between groups in terms of their follow up were adequately described and analyzed | Participants analyzed in the groups to which they were randomized | Outcomes were measured in the same way for treatment groups | Outcomes were measured in a reliable way | Appropriate statistical analysis. was used | Trial design was appropriate, and any deviations from the standard RCT design (individual randomization, parallel groups) accounted for in the conduct and analysis of the trial | Total Number of Criteria | Number of Criteria Met | Number of Criteria Not Met | Number of Criteria Unclear | Number of Criteria Not Applicable | Source of the opinion clearly identified | The stated position is the result of an analytical process and there is logic in the opinion expressed | Any incongruence with the literature/sources is logically defended | Number of Criteria Met | Number of Criteria Unclear | Number of Criteria Not Met |  |
| Patel et al. (2010) | RCT | Y | NA | Y | NA | NA | Y | Y | Y | Y | Y | Y | Y | Y | 13 | 10 | 0 | 0 | 3 | 1 | 1 | 1 | 3 | 0 | 0 | Include |
| Patel et al. (2011) | RCT | Y | NA | Y | NA | NA | Y | Y | Y | Y | Y | Y | Y | Y | 13 | 10 | 0 | 0 | 3 | 1 | 1 | 1 | 3 | 0 | 0 | Include |
| Dawson et al. (2016) | RCT | Y | Y | Y | NA | NA | Y | NA | Y | NA | Y | Y | Y | Y | 13 | 9 | 0 | 0 | 4 | 1 | 1 | 1 | 3 | 0 | 0 | Include |
| Indu et al. (2018) | RCT | Y | Y | NA | NA | Y | Y | Y | Y | Y | Y | Y | Y | Y | 13 | 11 | 0 | 0 | 2 | 1 | 1 | 1 | 3 | 0 | 0 | Include |
| Chowdhary et al. (2016) | Pilot RCT preceded by intervention development | Y | U | Y | U | NA | U | Y | Y | Y | Y | Y | Y | Y | 13 | 9 | 0 | 3 | 1 | 1 | 1 | 1 | 3 | 0 | 0 | Include |
| Tomlinson et al. (2015) | RCT | Y | U | Y | NA | NA | Y | Y | Y | U | Y | Y | Y | Y | 13 | 9 | 0 | 2 | 2 | 1 | 1 | 1 | 3 | 0 | 0 | Include |
| Rahman et al. (2008) | RCT | Y | Y | Y | Y | U | Y | Y | Y | U | Y | Y | Y | Y | 13 | 11 | 0 | 2 | 0 | 1 | 1 | 1 | 3 | 0 | 0 | Include |
| Matsuzaka et al. (2017) | RCT | Y | Y | Y | NA | NA | Y | Y | Y | Y | Y | Y | Y | Y | 13 | 11 | 0 | 0 | 2 | 1 | 1 | 1 | 3 | 0 | 0 | Include |

Note: Y = yes, N = no, U = unclear, NA = not applicable.

*Quasi-experimental studies*

| Author(s) and Year | Critical Appraisal Tool Type and Corresponding Criteria for Study | | | | | | | | | | Summary of Critical Appraisal for Study | | | | | Criteria from the Text & Opinion Critical Appraisal Tool Used | | | Summary of Critical Appraisal for Discussion | | | Overall Appraisal |
| --- | --- | --- | --- | --- | --- | --- | --- | --- | --- | --- | --- | --- | --- | --- | --- | --- | --- | --- | --- | --- | --- | --- |
|  | Critical Appraisal Tool Used (Additional Details) | The 'cause' and the 'effect' are clear (i.e. there is no confusion about which variable comes first) | Participants included in any comparisons are similar | The participants included in any comparisons received similar treatment/care, other than the exposure or intervention of interest | There is a control group | There are multiple measurements of the outcome both pre and post the intervention/exposure | Follow up is complete and if not, were differences between groups in terms of their follow up are adequately described and analyzed | The outcomes of participants are included in any comparisons measured in the same way | Outcomes are measured in a reliable way | Appropriate statistical analysis was used | Total Number of Criteria | Number of Criteria Met | Number of Criteria Not Met | Number of Criteria Unclear | Number of Criteria Not Applicable | Source of the opinion clearly identified | The stated position is the result of an analytical process and there is logic in the opinion expressed | Any incongruence with the literature/sources is logically defended | Number of Criteria Met | Number of Criteria Unclear | Number of Criteria Not Met |  |
| Maulik et al. (2017) | Quasi-experimental (pre-post) | Y | NA | NA | NA | Y | Y | Y | Y | Y | 9 | 6 | 0 | 0 | 3 | 1 | 1 | 1 | 3 | 0 | 0 | Include |
| Sibeko et al. (2018) | Quasi-experimental (pre-post) | Y | Y | Y | NA | Y | Y | Y | Y | Y | 9 | 8 | 0 | 0 | 1 | 1 | 1 | 1 | 3 | 0 | 0 | Include |
| Murray et al. (2014) | Quasi-Experimental (process evaluation) | Y | Y | Y | NA | NA | Y | Y | Y | Y | 9 | 7 | 0 | 0 | 2 | 1 | 1 | 1 | 3 | 0 | 0 | Include |
| Chibanda et al. (2011) | Quasi-experimental (pre-post) | Y | Y | Y | NA | N | Y | Y | Y | Y | 9 | 7 | 1 | 0 | 1 | 1 | 1 | 1 | 3 | 0 | 0 | Include |
| O'Donnell et al. (2014) | Quasi-Experimental (pre-post) | Y | Y | N | N | Y | Y | Y | Y | Y | 9 | 7 | 2 | 0 | 0 | 1 | 1 | 1 | 3 | 0 | 0 | Include |
| Petersen et al. (2012b) | Quasi-Experimental + Process Evaluation | Y | Y | Y | Y | Y | Y | Y | Y | Y | 9 | 9 | 0 | 0 | 0 | 1 | 1 | 1 | 3 | 0 | 0 | Include |

Note: Y = yes, N = no, U = unclear, NA = not applicable.

*Cohort studies*

| Author(s) and Year | Critical Appraisal Tool Type and Corresponding Criteria for Study | | | | | | | | | | | | Summary of Critical Appraisal for Study | | | | | Criteria from the Text & Opinion Critical Appraisal Tool Used | | | Summary of Critical Appraisal for Discussion | | | Overall Appraisal |
| --- | --- | --- | --- | --- | --- | --- | --- | --- | --- | --- | --- | --- | --- | --- | --- | --- | --- | --- | --- | --- | --- | --- | --- | --- |
|  | Critical Appraisal Tool Used (Additional Details) | The two groups are similar and recruited from the same population | The exposures were measured similarly to assign people to both exposed and unexposed groups | The exposure was measured in a valid and reliable way | Confounding factors are identified | Strategies to deal with confounding factors are stated | The groups/participants were free of the outcome at the start of the study (or at the moment of exposure) | Outcomes were measured in a valid and reliable way | The follow up time is reported and sufficient to be long enough for outcomes to occur | Follow up was complete, and if not, the reasons to loss to follow up are described and explored | Strategies to address incomplete follow up are utilized | Appropriate statistical analysis was used | Total Number of Criteria | Number of Criteria Met | Number of Criteria Not Met | Number of Criteria Unclear | Number of Criteria Not Applicable | Source of the opinion clearly identified | The stated position is the result of an analytical process and there is logic in the opinion expressed | Any incongruence with the literature/sources is logically defended | Number of Criteria Met | Number of Criteria Unclear | Number of Criteria Not Met |  |
| Selohilwe et al. (2019) | Cohort (1-arm) & Process Evaluation | NA | NA | Y | Y | NA | Y | Y | Y | Y | U | Y | 11 | 7 | 0 | 1 | 3 | 1 | 1 | 1 | 3 | 0 | 0 | Include |
| Jordans et al.  (2017) | Cohort | Y | Y | Y | NA | NA | Y | Y | Y | Y | NA | Y | 11 | 8 | 0 | 0 | 3 | 1 | 1 | 1 | 3 | 0 | 0 | Include |

Note: Y = yes, N = no, U = unclear, NA = not applicable.

*Analytical cross-sectional studies*

| Author(s) and Year | Critical Appraisal Tool Type and Corresponding Criteria for Study | | | | | | | | | Summary of Critical Appraisal for Study | | | | | Criteria from the Text & Opinion Critical Appraisal Tool Used | | | Summary of Critical Appraisal for Discussion | | | Overall Appraisal |
| --- | --- | --- | --- | --- | --- | --- | --- | --- | --- | --- | --- | --- | --- | --- | --- | --- | --- | --- | --- | --- | --- |
|  | Critical Appraisal Tool Used (Additional Details) | The criteria for inclusion in the sample are clearly defined | The study subjects and the setting are described in detail | The exposure was measured in a valid and reliable way | Objective, standard criteria were used for measurement of the condition | Confounding factors were identified | Strategies to deal with confounding factors are stated | The outcomes were measured in a valid and reliable way | Appropriate statistical analysis was used | Total Number of Criteria | Number of Criteria Met | Number of Criteria Not Met | Number of Criteria Unclear | Number of Criteria Not Applicable | Source of the opinion clearly identified | The stated position is the result of an analytical process and there is logic in the opinion expressed | Any incongruence with the literature/sources is logically defended | Number of Criteria Met | Number of Criteria Unclear | Number of Criteria Not Met |  |
| Fils-Aimé et al. (2018) | Analytical Cross-sectional | Y | Y | Y | Y | NA | NA | Y | Y | 8 | 6 | 0 | 0 | 2 | 1 | 1 | 1 | 3 | 0 | 0 | Include |
| Khoja et al. (2016) | Analytical Cross-sectional (some qualitative process data) | Y | Y | Y | Y | NA | NA | Y | Y | 8 | 6 | 0 | 0 | 2 | 1 | 1 | 1 | 3 | 0 | 0 | Include |

Note: Y = yes, N = no, U = unclear, NA = not applicable.
